# Supplementary material for: Evolution of antibiotic resistance at low antibiotic concentrations including selection below the minimal selective concentration
Source: Commun Biol. 2020 Sep 3;3:467. doi: 10.1038/s42003-020-01176-w (PMC7471295; doi:10.1038/s42003-020-01176-w)
Supplement: Supplementary file 1 — Supplementary Information [file 42003_2020_1176_MOESM1_ESM.pdf]

Supplementary Information

Supplementary Figures:

Assessing the selective potential of macrolide antibiotics – Range finding experiments (low concentrations)

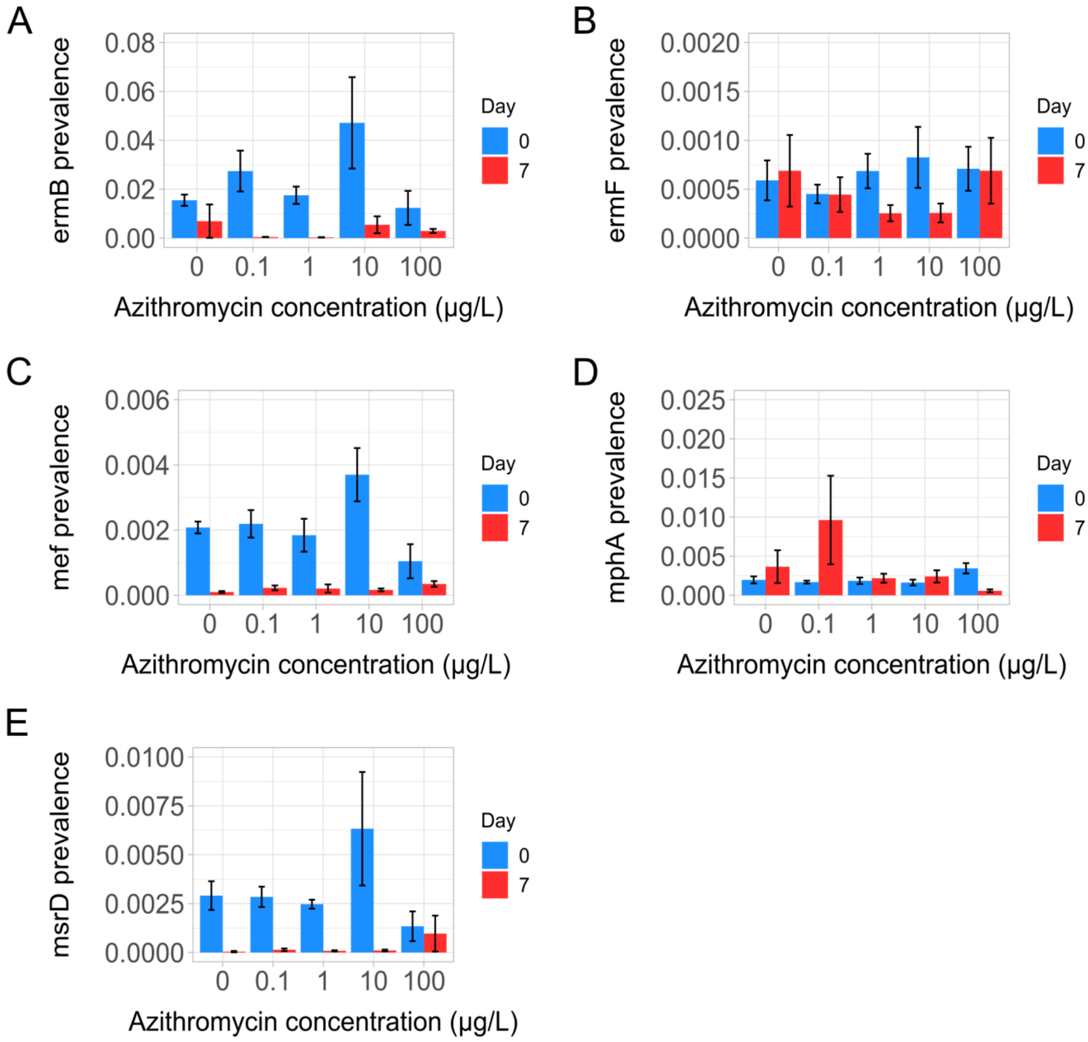

Supplementary Figure 1: Macrolide resistance genes as a function of low concentrations of azithromycin. A: *ermB,* B: *ermF,* C: *mef* family (one high, outlier replicated has been removed from the 100 µg/L sample), D: *mphA,* E: *msrD*. No positive selection is observed at any azithromycin concentration tested for any of the genes tested. Error bars represent the standard error.

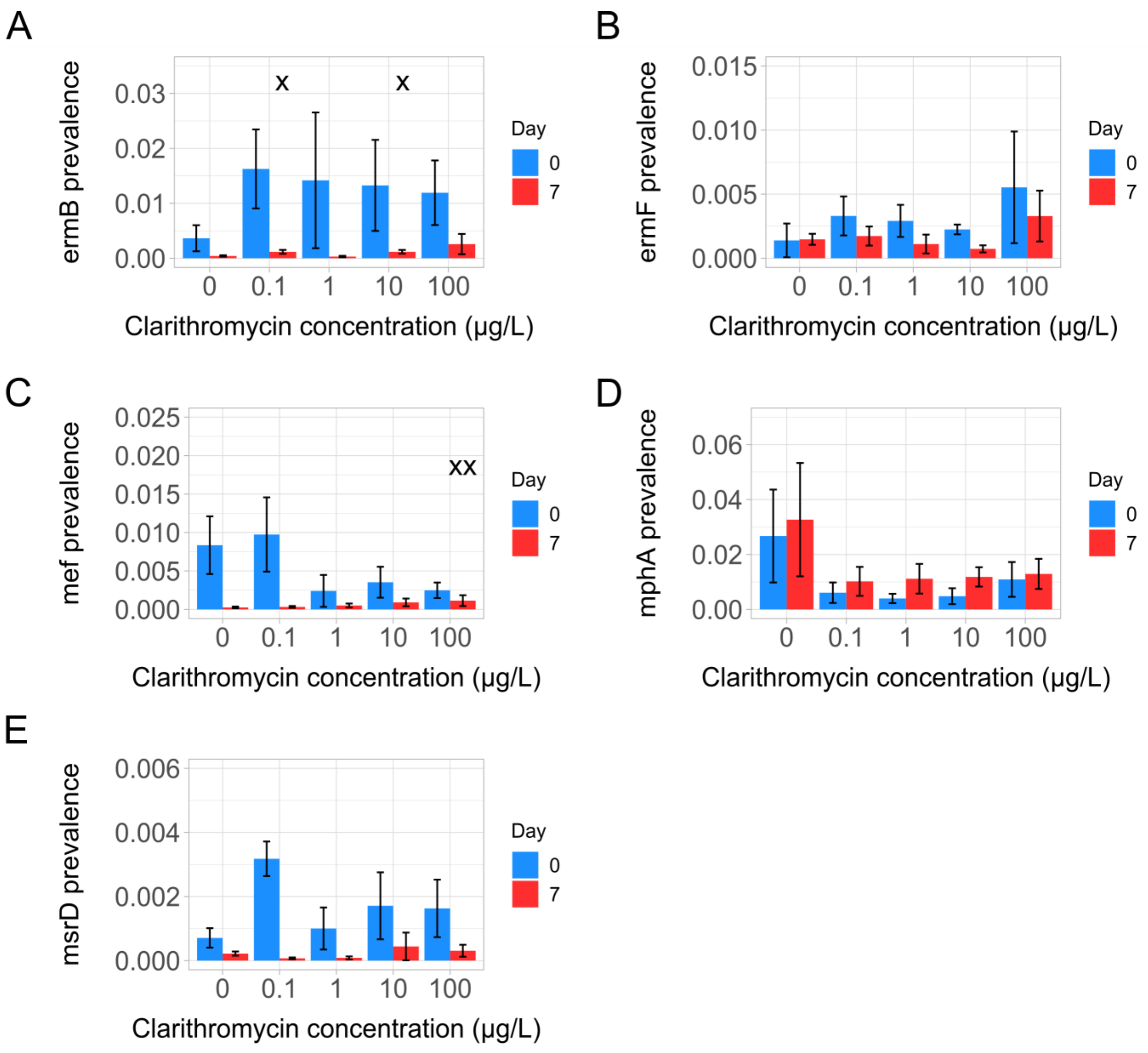

Supplementary Figure 2: Macrolide resistance genes as a function of low concentrations of clarithromycin. A: *ermB,* B: *ermF,* C: *mef* family, D: *mphA,* E: *msrD*. No positive selection is observed at any clarithromycin concentration tested for any of the genes tested. Significant increase of *ermB* in comparison to the no antibiotic control is observed to 90% at 0.1 and 10 µg/L of clarithromycin. This is not above the starting prevalence. A significant increase in *mef* family prevalence, in comparison to the no antibiotic control, is observed to 95% confidence at 100 µg/L of clarithromycin. This is not above the starting prevalence. One outlier replicate has been removed from the 0 ug/L at day 0. Error bars represent the standard error. x = an increase in prevalence, to 90% confidence, in comparison to the no antibiotic control but not increasing over the starting prevalence. xx = an increase in prevalence, to 95% confidence, in comparison to the no antibiotic control but not increasing over the starting prevalence.

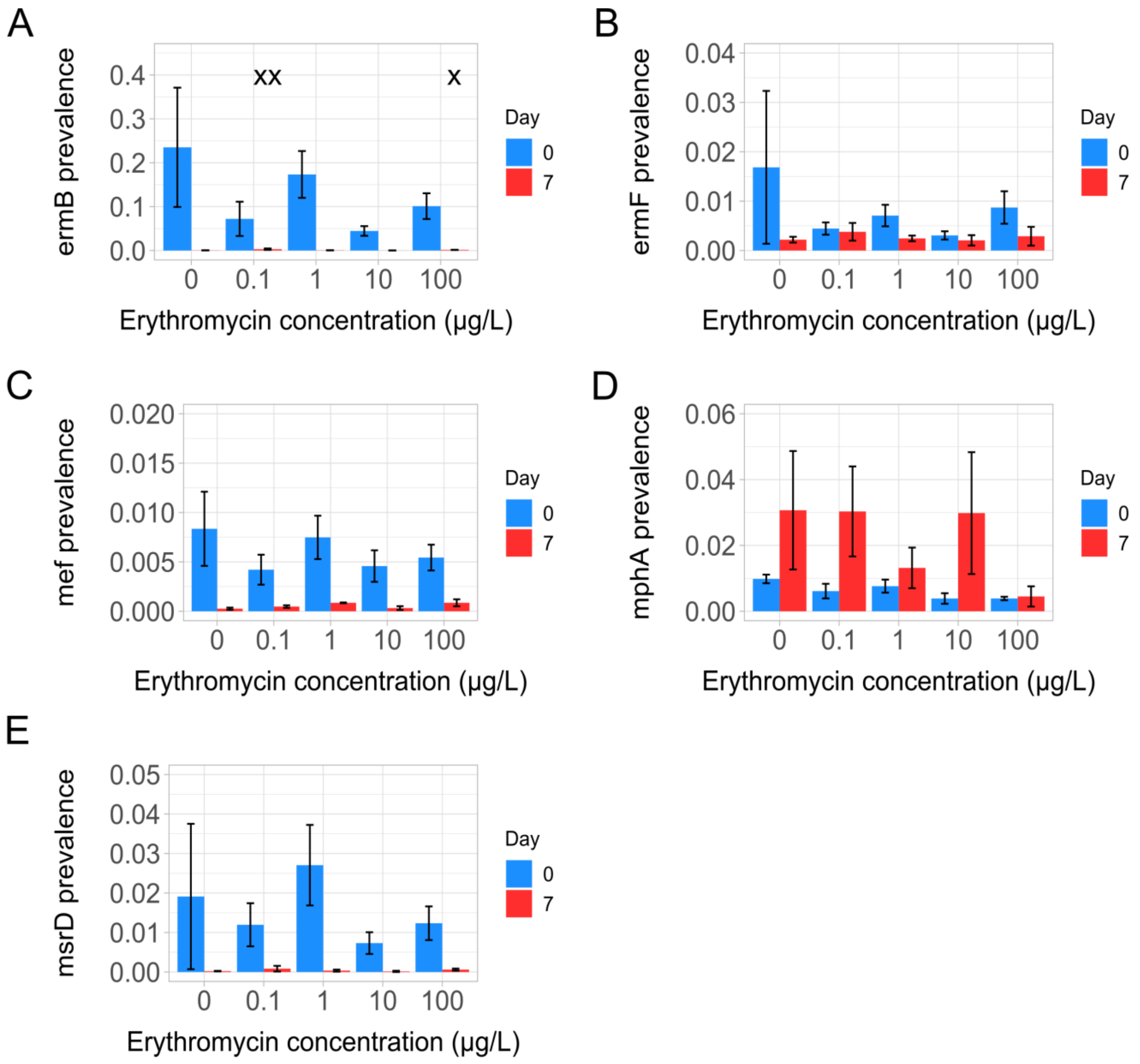

Supplementary Figure 3: Macrolide resistance genes as a function of low concentrations of erythromycin. A: *ermB,* B: *ermF,* C: *mef* family, D: *mphA,* E: *msrD*. No positive selection is observed at any erythromycin concentration tested for any of the genes tested. Significant increase of *ermB* in comparison to the no antibiotic control is observed to 90% at 10 µg/L and to 95% confidence at 0.1 µg/L of erythromycin. This is not above the starting prevalence. One outlier replicate has been removed from the 0 ug/L at day 0. Error bars represent the standard error. x = an increase in prevalence, to 90% confidence, in comparison to the no antibiotic control but not increasing over the starting prevalence. xx = an increase in prevalence, to 95% confidence, in comparison to the no antibiotic control but not increasing over the starting prevalence.

Assessing the selective potential of macrolide antibiotics – Range finding experiments (high concentrations)

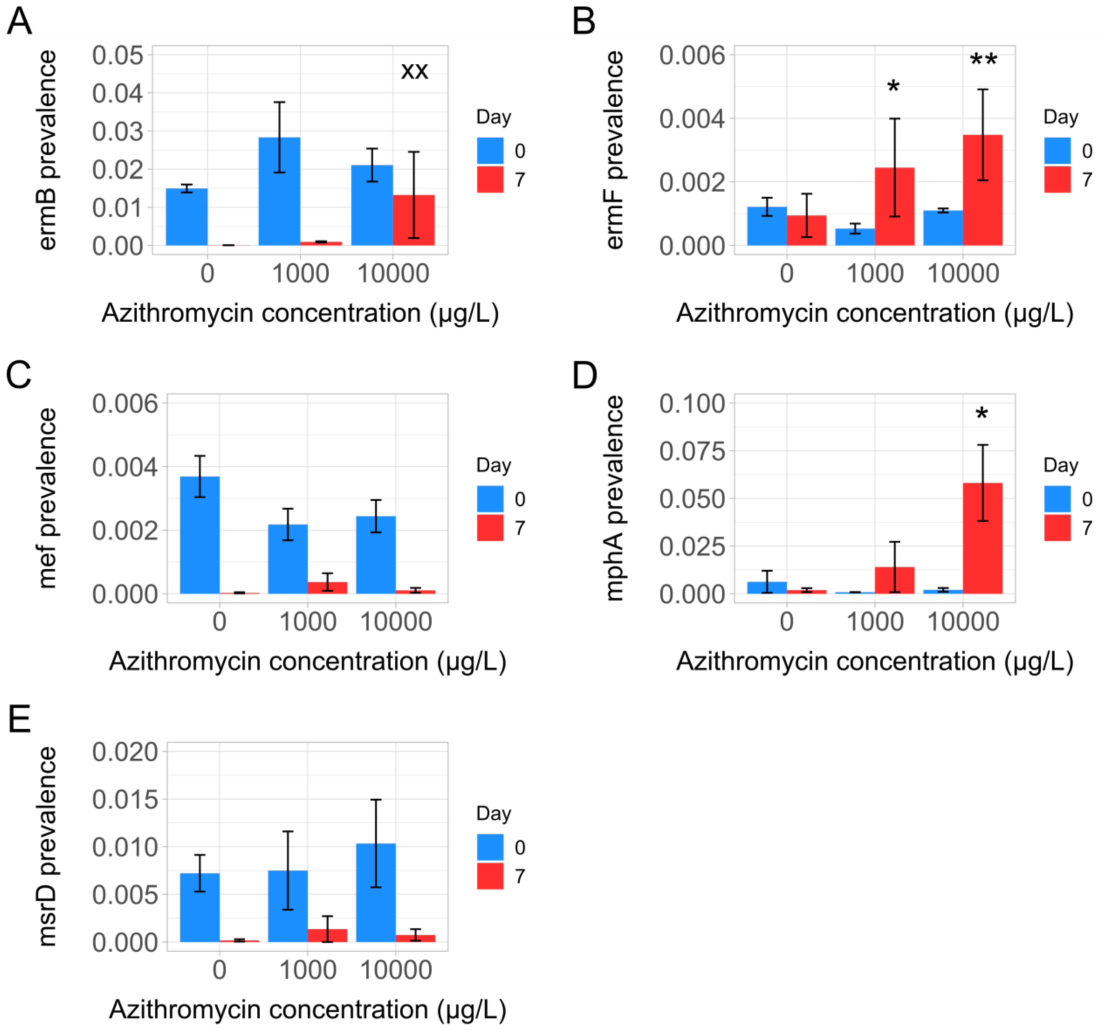

Supplementary Figure 4: Macrolide resistance genes as a function of high azithromycin concentrations A: *ErmB*. Only increased in prevalence in comparison to the no antibiotic control at 10,000 µg/L (p = 0.007, Dunn's test) although this is not higher than the starting prevalence. B: *ErmF.* Positive selection is observed at 1,000 µg/L to 90% confidence (p = 0.0784, Dunn's test) and at 10,000 µg/L to 95% confidence (p = 0.0368, Dunn's test). C: *Mef* family. No positive selection is occurring at any concentration. D: *MphA*. Positive selection is only observed at 10,000 µg/L (p = 0.0368, Dunn's test). E: *MsrD*. Significant positive selection is not observed for *msrD*. x = an increase in prevalence, to 90% confidence, in comparison to the no antibiotic control but not increasing over the starting prevalence. xx = an increase in prevalence, to 95% confidence, in comparison to the no antibiotic control but not increasing over the starting prevalence. * = significant positive selection to 90% confidence, ** = significant positive selection to 95% confidence. Error bars represent the standard error.

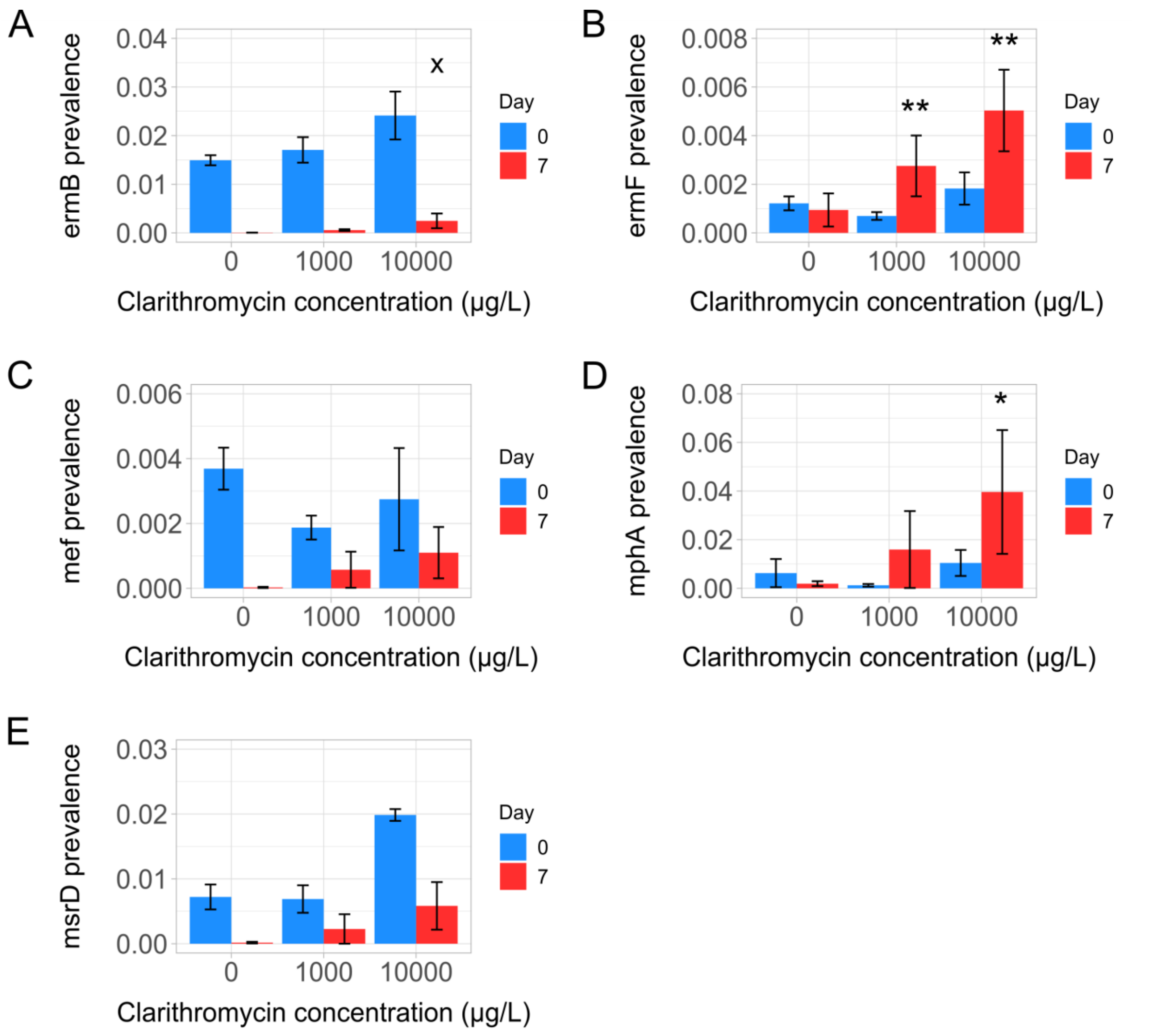

Supplementary Figure 5: Macrolide resistance genes as a function of high clarithromycin concentrations A: *ErmB*. Only a significant increase in prevalence, in comparison to the no antibiotic control, is occurring at 10,000 μg/L (p = 0.0104, Dunn's test) although this is not higher than the starting prevalence. B: *ErmF.* For *ermF* positive section is observed at 1,000 and 10,000 μg/L (p = 0.00953 and 0.00375, respectively, GLM (Gamma, log)). C: *Mef* family. No positive selection is occurring at any concentration. D: *MphA*. Significant selection is only seen for *mphA* (to 90% confidence) at a clarithromycin concentration of 10,000 μg/L (p = 0.068, Dunn's test).E: *MsrD*. Significant positive selection is not observed for *msrD*. x = an increase in prevalence, to 90% confidence, in comparison to the no antibiotic control but not increasing over the starting prevalence. * = significant positive selection to 90% confidence, ** = significant positive selection to 95% confidence. Error bars represent the standard error.

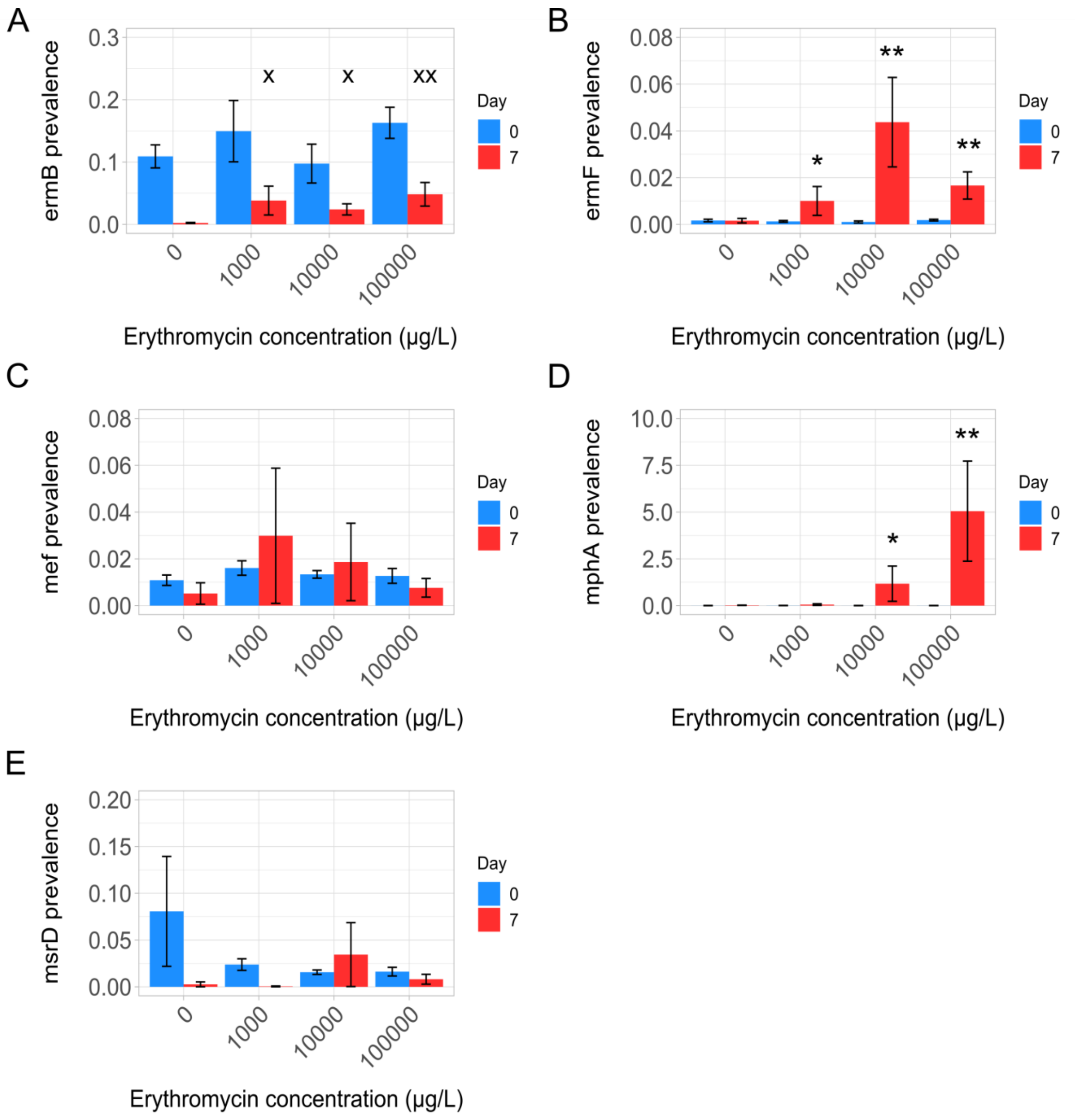

Supplementary Figure 6: Macrolide resistance genes as a function of high erythromycin concentrations A: *ErmB*. Only a significant increase in prevalence is seen for *ermB.* Here, it is observed at 1,000, 10,000 and 100,000 µg/L (*p* = 0.0705, 0.0565 and 0.0157, Dunn's test) of erythromycin. This is not over the starting prevalence. B: *ErmF.* For, *ermF,* a significant increase in prevalence in observed to 90% confidence at 1,000 µg/L and to 95% confidence at 10,000 and 100,000 µg/L (*p* = 0.0108 and 0.0163, Dunn's test) of erythromycin. C: *Mef* family. No positive selection is occurring at any concentration. D: *MphA*. Significant selection of *mphA* is seen to 90% confidence at 10,000 (*p* = 0.068, Dunn's test) and to 95% confidence at 100,000 µg/L (*p* = 0.0023, Dunn's test). E: *MsrD*. Significant positive selection is not observed for *msrD*. x = an increase in prevalence, to 90% confidence, in comparison to the no antibiotic control but not increasing over the starting prevalence. xx = an increase in prevalence, to 95% confidence, in comparison to the no antibiotic control but not

increasing over the starting prevalence. * = significance to 90% confidence, ** = significance to 95% confidence. Error bars represent the standard error.

Final macrolide experiment

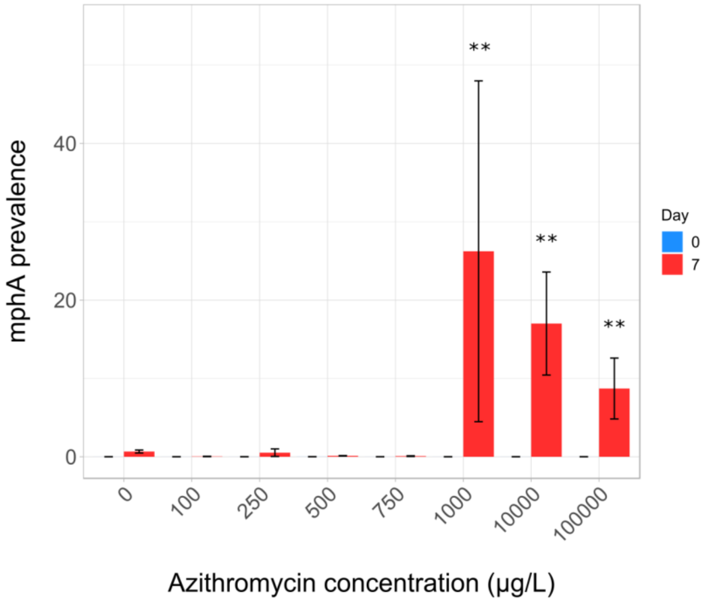

Supplementary Figure 7: *mphA* selection at a range of azithromycin concentrations. Significant selection is seen for *mphA* at 1,000, 10,000 and 100,000 µg/L (*p* = 9.21E-5, 0.000413 and 0.003762, respectively, GLM (Gamma, log)) of azithromycin. Error bars represent standard error. ** = significant positive selection to 95% confidence.

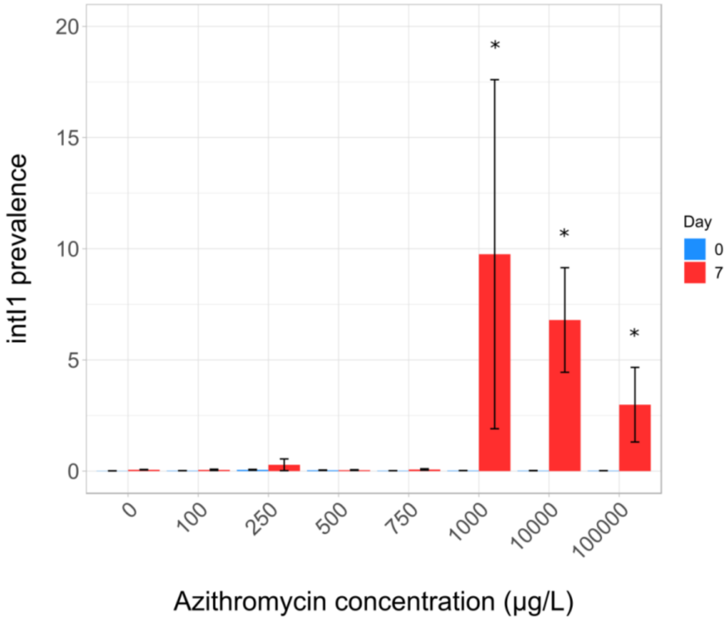

Supplementary Figure 8: *intI1* selection at a range of azithromycin concentrations. Positive selection for *intI1* was observed to 90% confidence at 1,000, 10,000 and 100,000 µg/L of

azithromycin (*p* = 0.0886, 0.0886 and 0.0932, respectively, GLM (Gamma, inverse)). * = significant positive selection to 90% confidence. Error bars represent standard error.

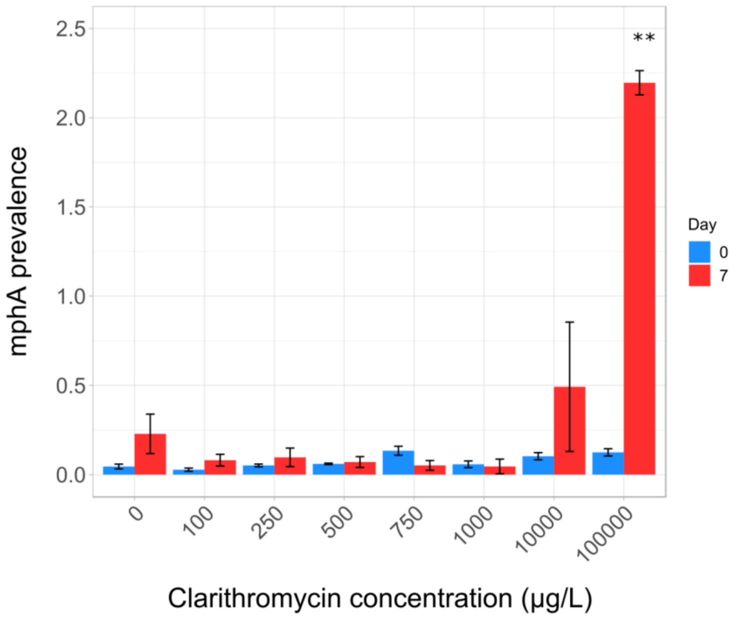

Supplementary Figure 9: *mphA* selection at a range of clarithromycin concentrations (one high outlier replicate removed from the no antibiotic control at day 7). Variation in prevalence was seen at day 0 (*p* = 0.001574, Kruskal Wallis) and the difference in prevalence between day 0 and 7 was, therefore, used for the statistical analysis. No significant selection is observed for most of the concentrations of clarithromycin tested. The only concentration where positive selection was seen was at 100,000 µg/L (*p* = 0.0446, Dunn's test (difference)). ** = significant positive selection to 95% confidence. Error bars represent standard error.

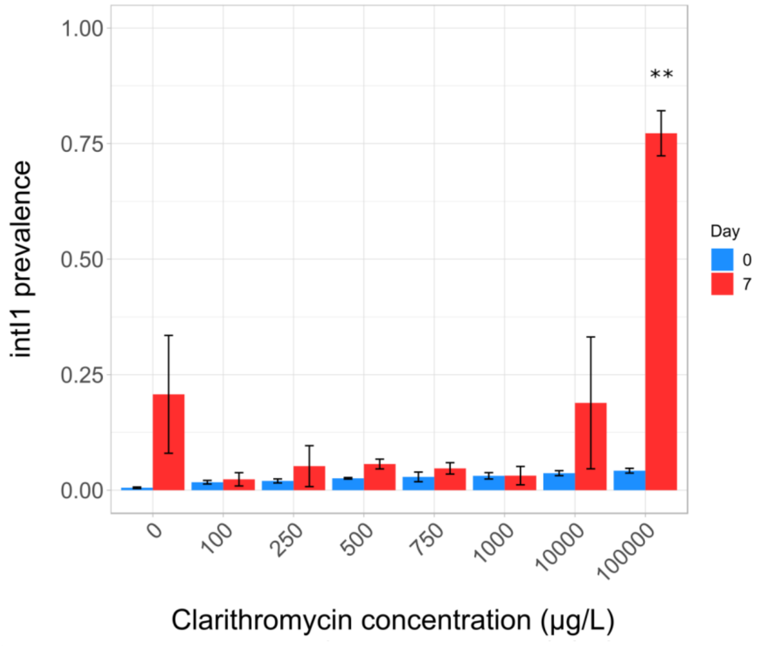

Supplementary Figure 10: *intI1* selection at a range of clarithromycin concentrations. Variation in the prevalence of *intI1* was observed at day 0 (p = 0.0018, Kruskal Wallis). Difference in prevalence was used, therefore, for the statistical analyses. No significant selection was observed until 100,000 µg/L of clarithromycin (p = 1.46e-05, Gaussian GLM (difference)). ** = significant positive selection to 95% confidence. Error bars represent standard error.

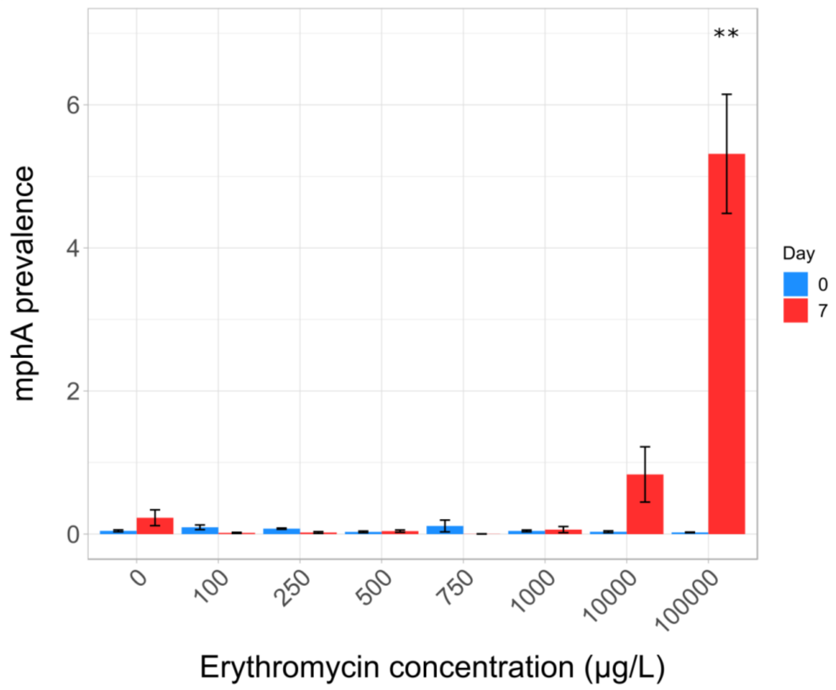

Supplementary Figure 11: *mphA* selection at a range of erythromycin concentrations (one high outlier replicate removed from the no antibiotic control at day 7). Significant selection is

first seen at 100,000 µg/L (p = 0.0361, Dunn's test). ** = significant positive selection to 95% confidence. Error bars show standard error.

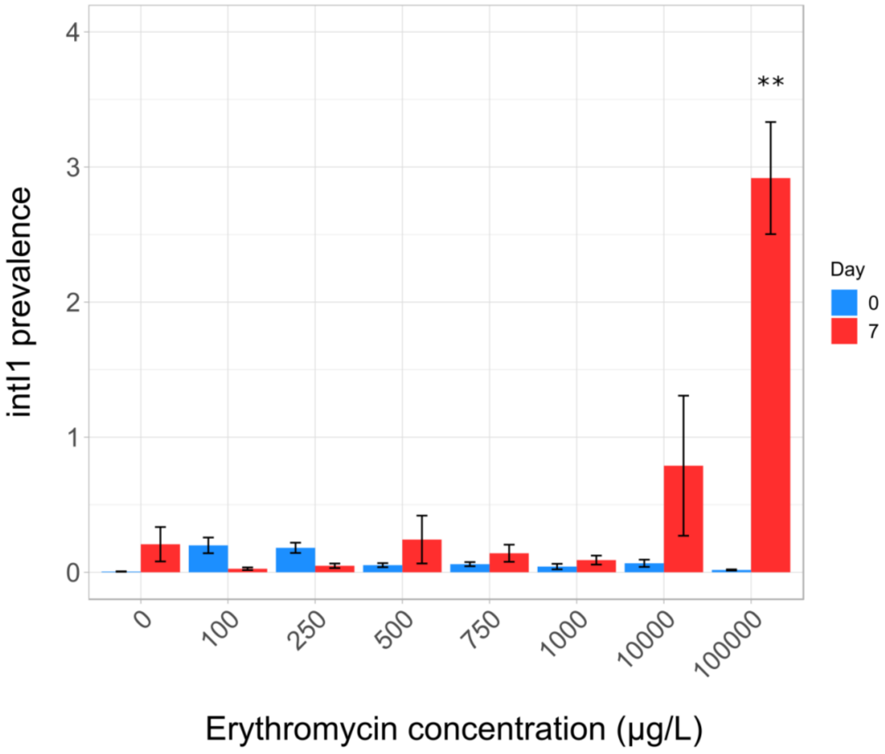

Supplementary Figure 12: *intI1* selection at a range of erythromycin concentrations. Variation of prevalence at day 0 was observed (p = 0.0002008, Kruskal Wallis). The prevalence was, therefore, used for statistical analyses. A significant increase in day 7 prevalence of *intI1* is observed only at 100,000 µg/L of erythromycin (p = 0.0142, Dunn's test (difference)). ** = significant positive selection to 95% confidence. Error bars show standard error.

Selection coefficients

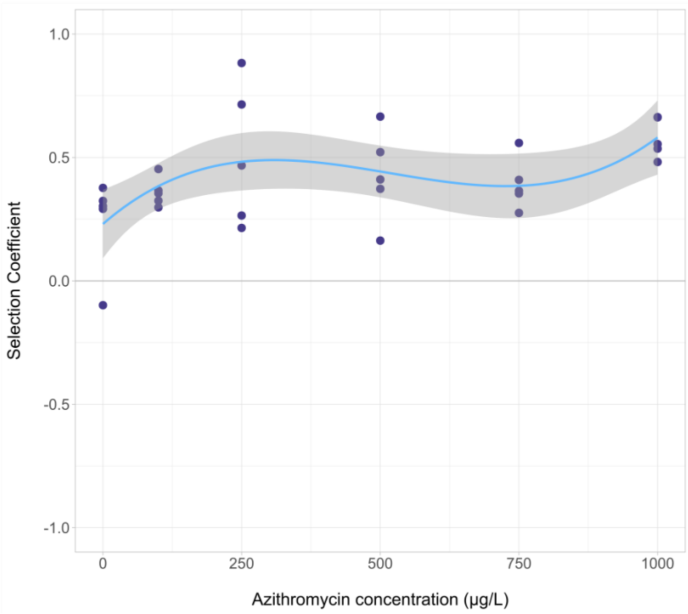

Supplementary Figure 13: Selection coefficient graph for the selection of *ermF* by azithromycin. The line of best fit (polynomial order 3, $y= 0.05846 + 0.0006282x – 2.2e^{-06}x^2 + 1.752e^{-09}x^3$, $R^2 = 0.07934$) never crosses the x axis and therefore a MSC cannot be determined.

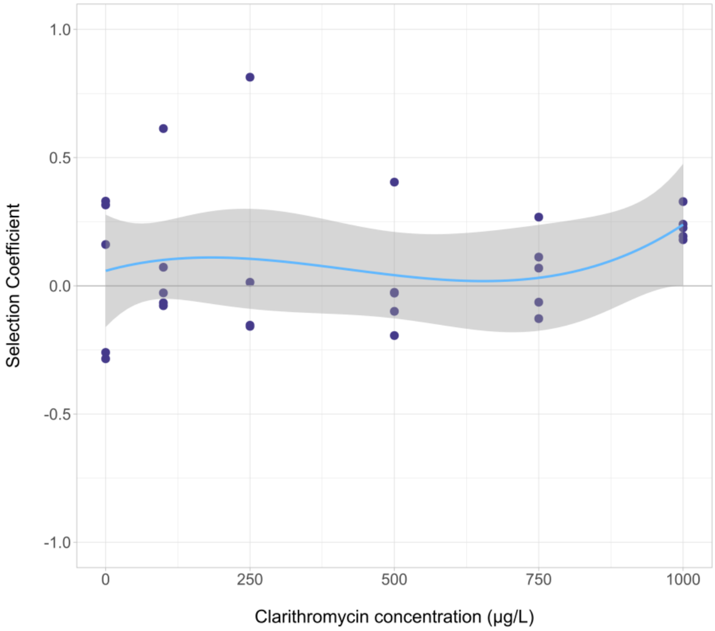

Supplementary Figure 14: Selection coefficient graph for the selection of *ermF* by clarithromycin. The line of best fit (polynomial order 3, $y = 0.2305 + 0.001955x – 4.514e^{-06}x^2 + 2.9e^{-09}x^3$, $R^2 = 0.3268$) never crosses the x axis and therefore a MSC cannot be determined.

Culture dependent assay

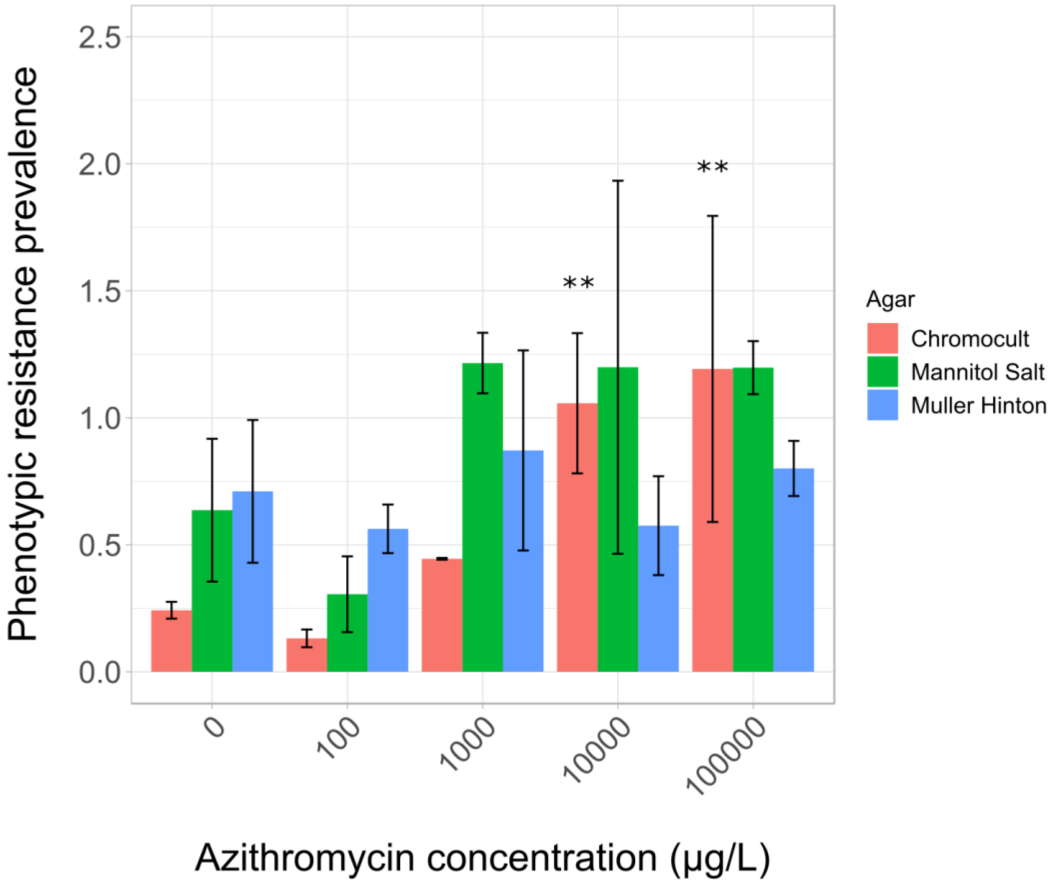

Supplementary Figure 15: Phenotypic resistance, plated on 3 agars, as a function of azithromycin concentration. No significant change in prevalence of resistance was observed for either mannitol salt agar or Muller Hinton agar at any concentration of azithromycin. On Chromocult agar, however, a significant increase was seen for resistant bacteria at 10,000 (p = 0.0493, Dunn's test) and 100,000 μg/L (p = 0.0493, Dunn's test) of azithromycin. ** = significance to 95% confidence. Error bars show standard error.

Metagenome analysis

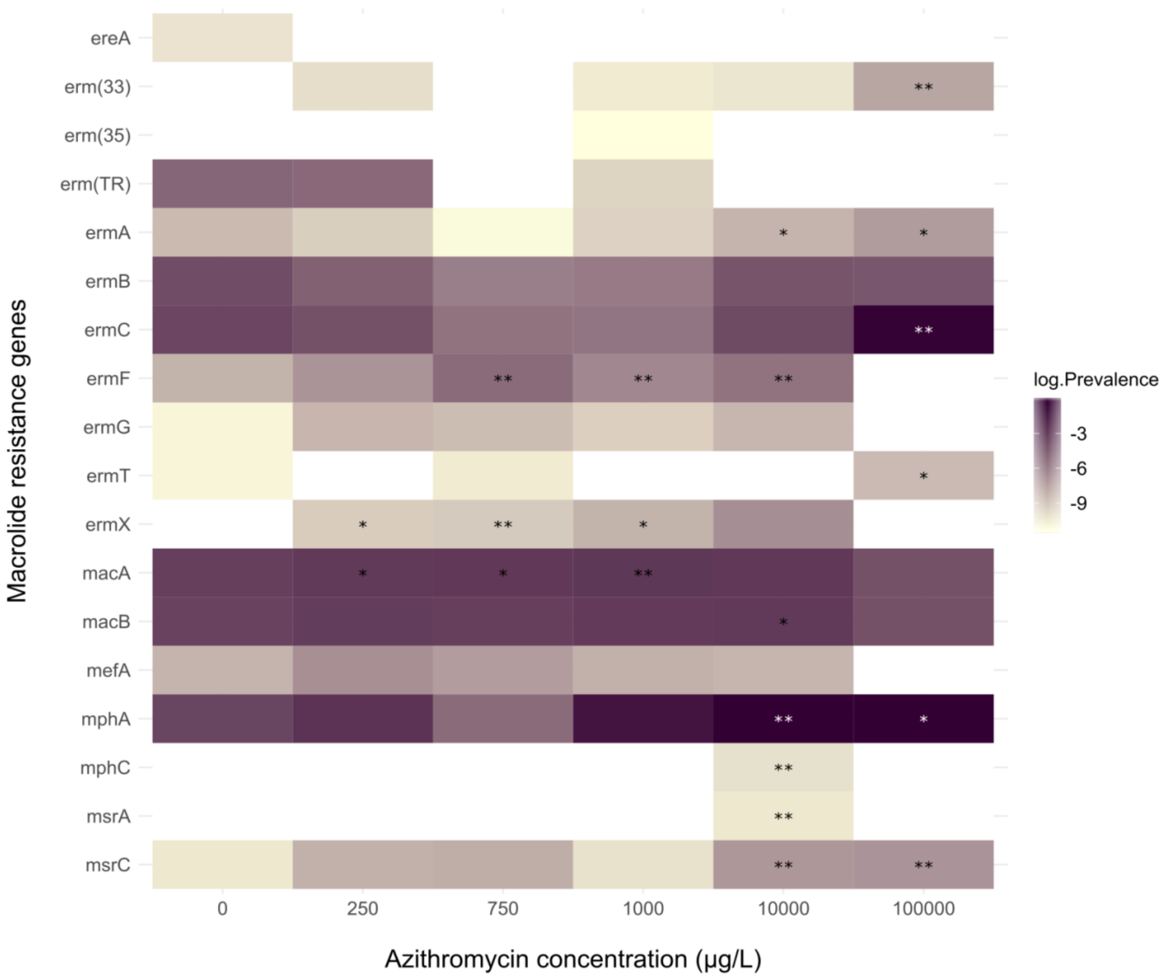

Supplementary Figure 16: Heatmap showing the change in prevalence of macrolide resistance genes as a function of azithromycin. The log value of the prevalence of genes (normalized to 16S rRNA copy number) is presented here. White areas represent where genes were below the detection limit. Genes that were below detection limit at every concentration of azithromycin are not included. * = significant to 90% confidence. ** = significance to 95% confidence.

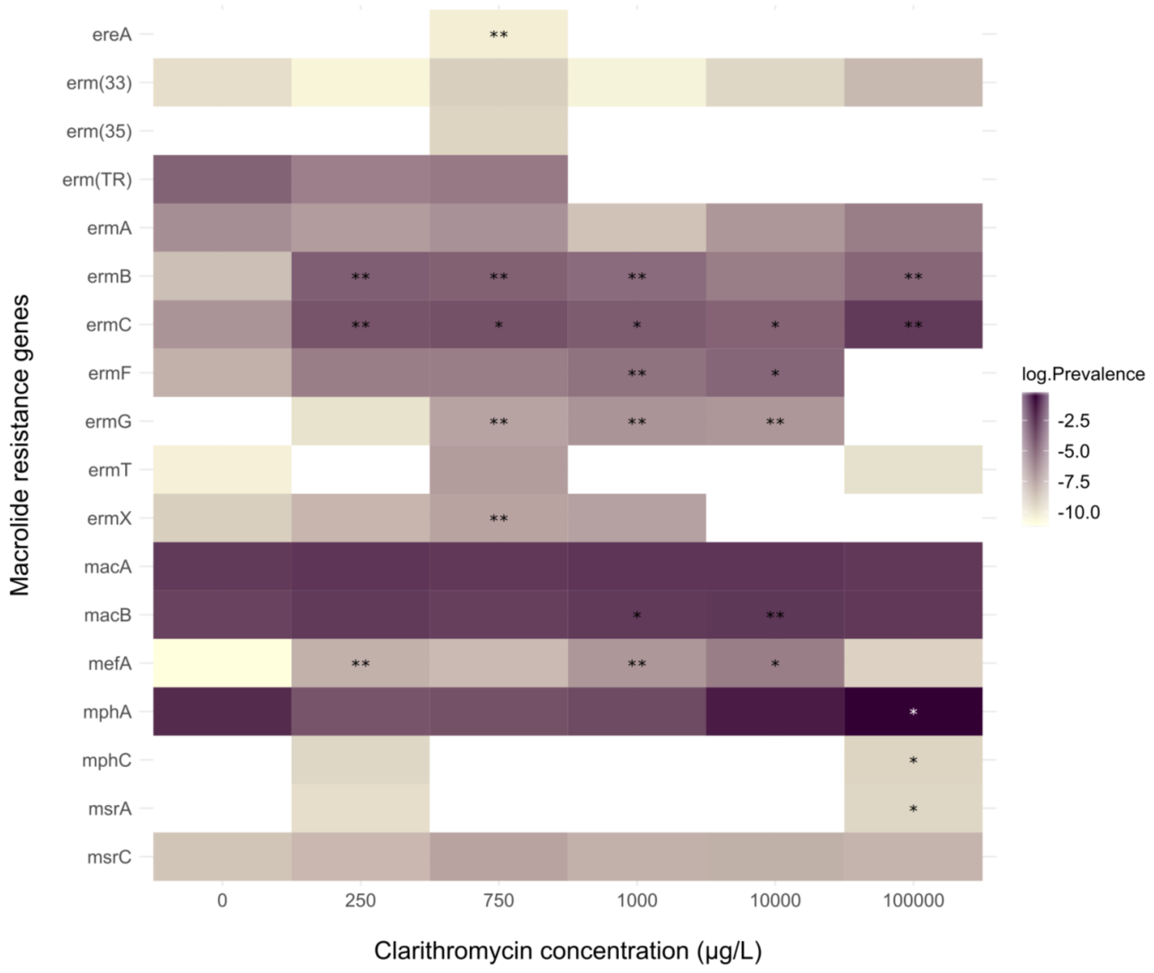

Supplementary Figure 17: Heatmap showing the change in prevalence of macrolide resistance genes as a function of clarithromycin. The log value of the prevalence of genes (normalized to 16S rRNA copy number) is presented here. White areas represent where genes were below the detection limit. Genes that were below detection limit at every concentration of clarithromycin are not included. * = significant to 90% confidence. ** = significance to 95% confidence.

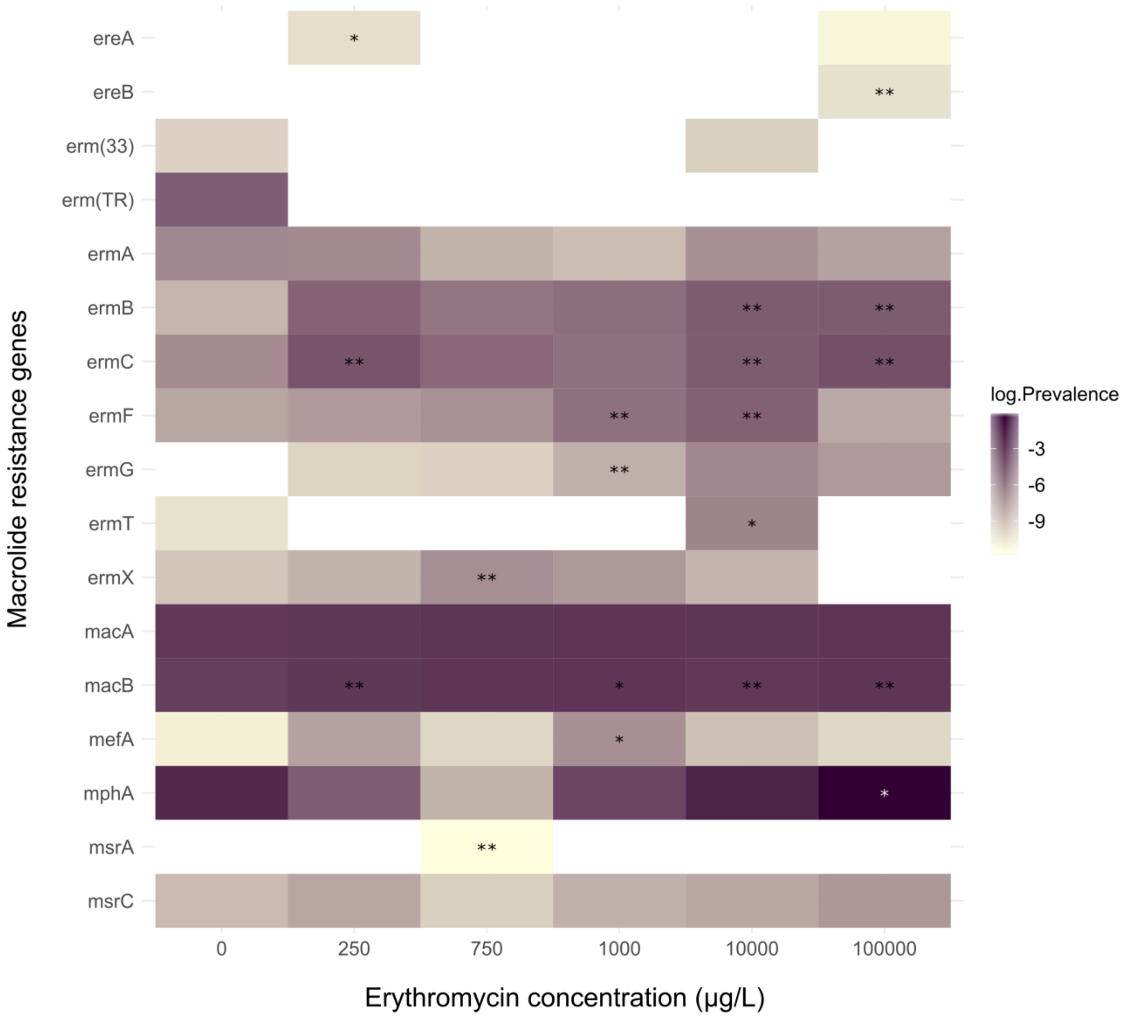

Supplementary Figure 18: Heatmap showing the change in prevalence of macrolide resistance genes as a function of erythromycin. The log value of the prevalence of genes (normalized to 16S rRNA copy number) is presented here. White areas represent where genes were below the detection limit. Genes that were below detection limit at every concentration of erythromycin are not included. * = significant to 90% confidence. ** = significance to 95% confidence.

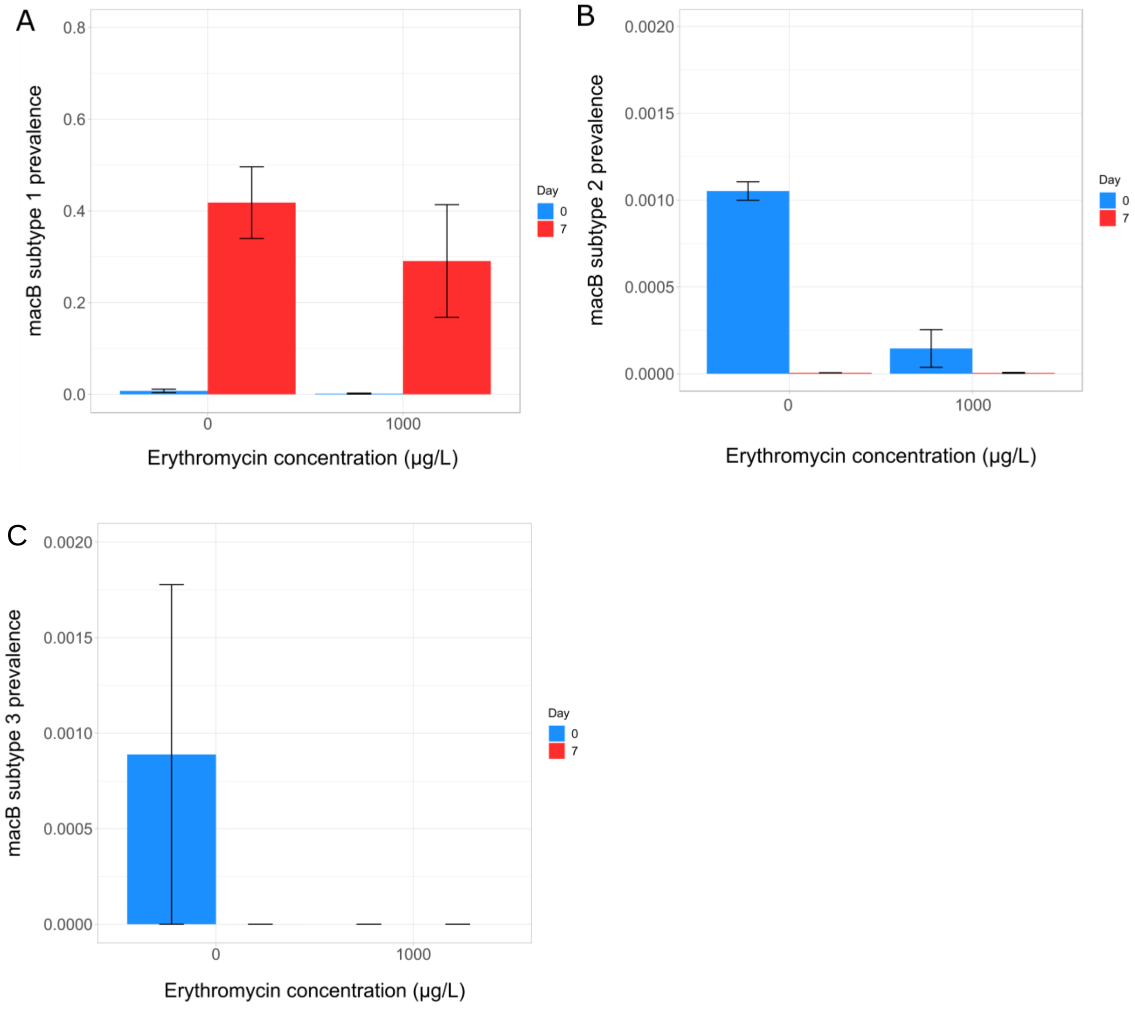

Supplementary Figure 19A: *macB* subtype 1, B: *macB* subtype 2 and C: *macB* subtype 3 as functions of erythromycin concentration. No significant positive selection is occurring for either *macB* subtype 1, 2 or 3 at 1,000 µg/L of erythromycin. Subtype 4 was undetectable in every sample tested. Error bars represent standard error.

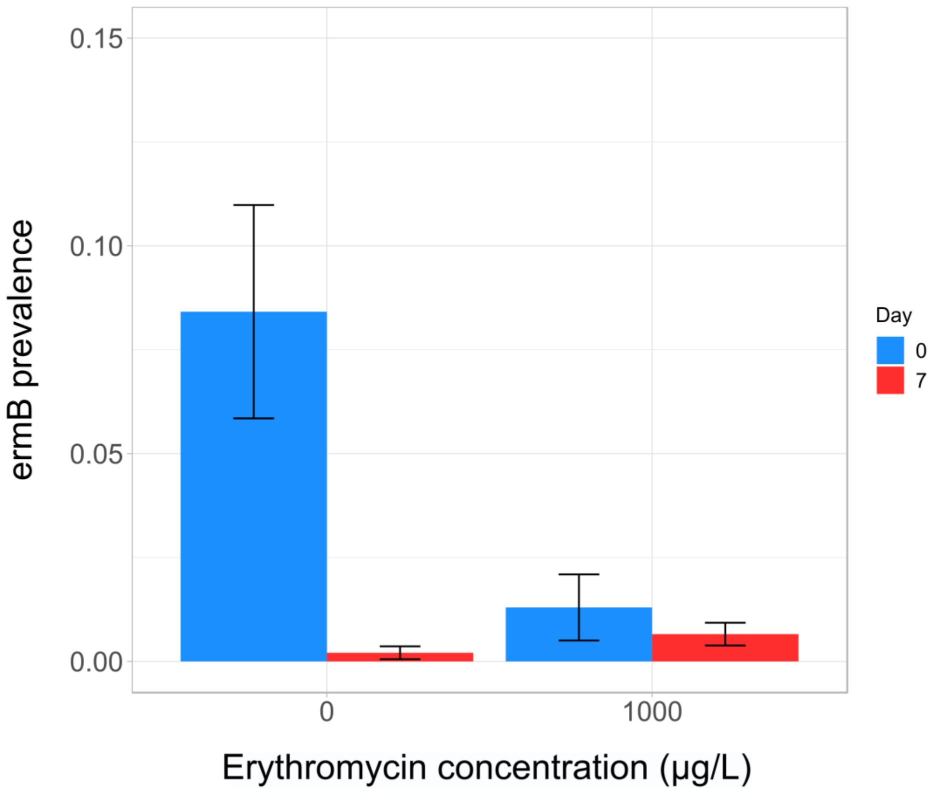

Supplementary Figure 20: *ermB* as a function of erythromycin concentration. No significant selection was observed for *ermB* in the presence of 1,000 µg/L compared to the no antibiotic control. Error bars represent standard error.

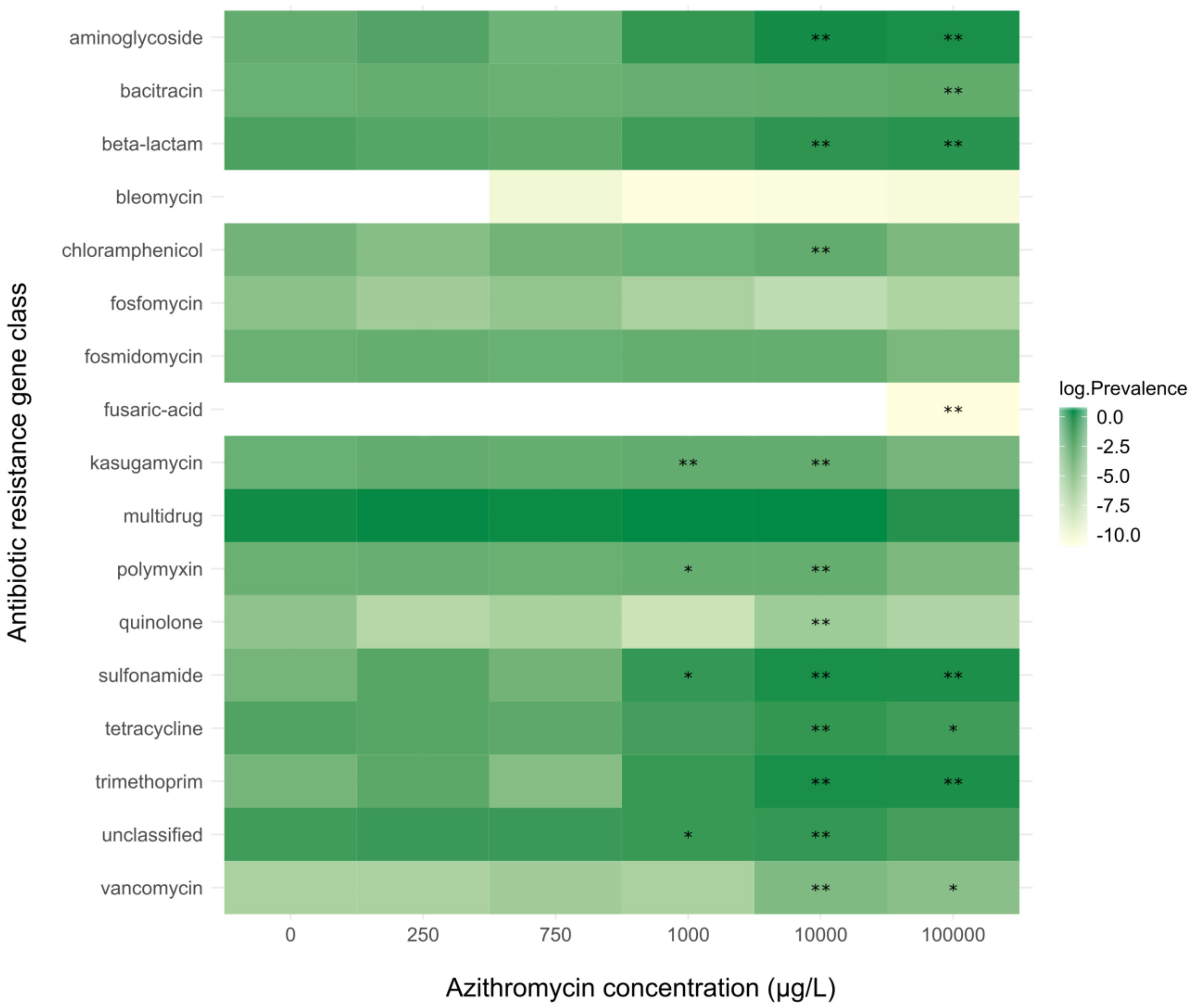

Supplementary Figure 21: Co-selection for antibiotic resistance gene classes by azithromycin. Log of the prevalence of resistance genes (normalized to 16S rRNA copy number) is represented here. * = significance to 90% confidence. ** = significance to 95% confidence.

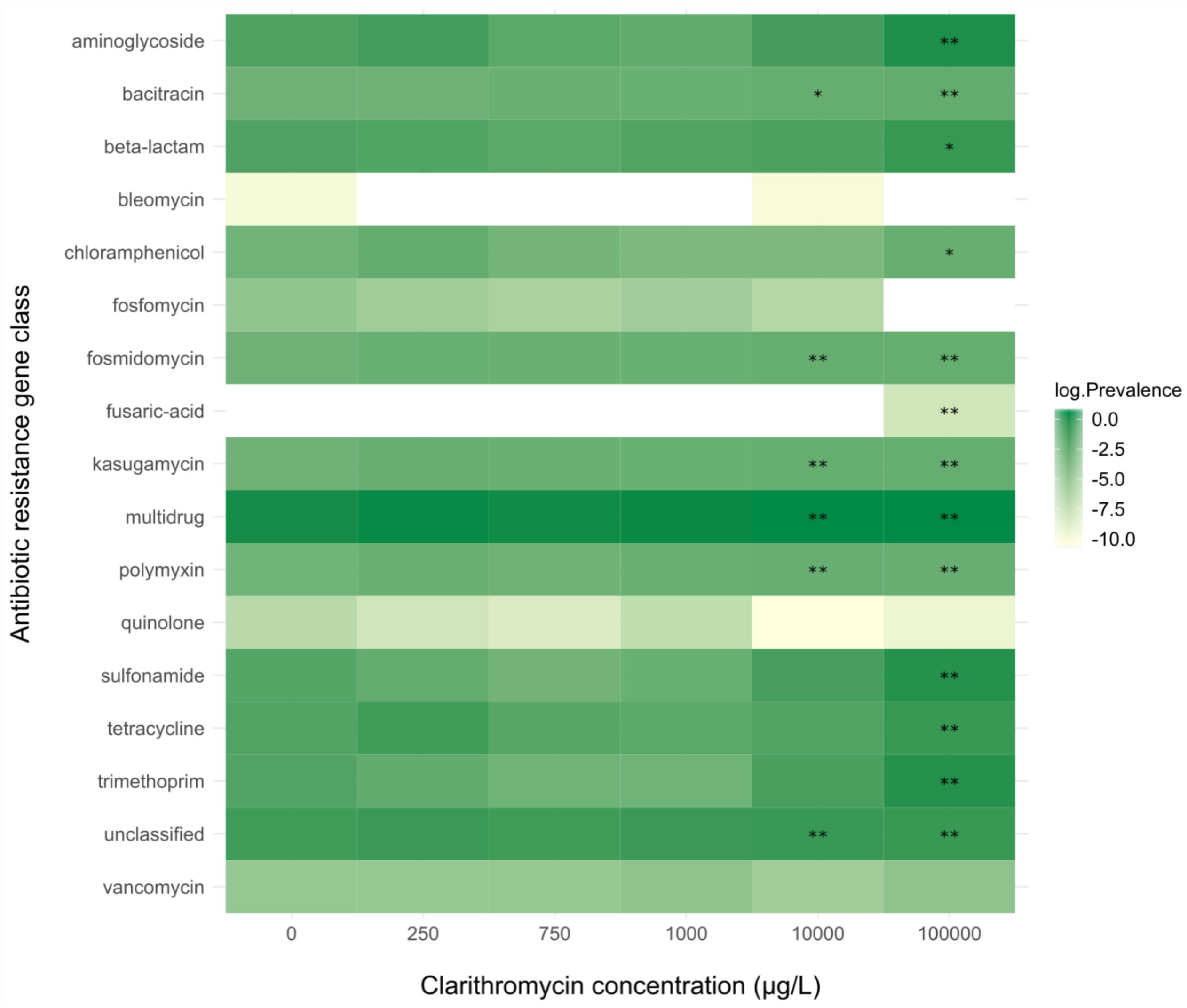

Supplementary Figure 22: Co-selection for antibiotic resistance gene classes by clarithromycin. Log of the prevalence of resistance genes (normalized to 16S rRNA copy number) is represented here. * = significance to 90% confidence. ** = significance to 95% confidence.

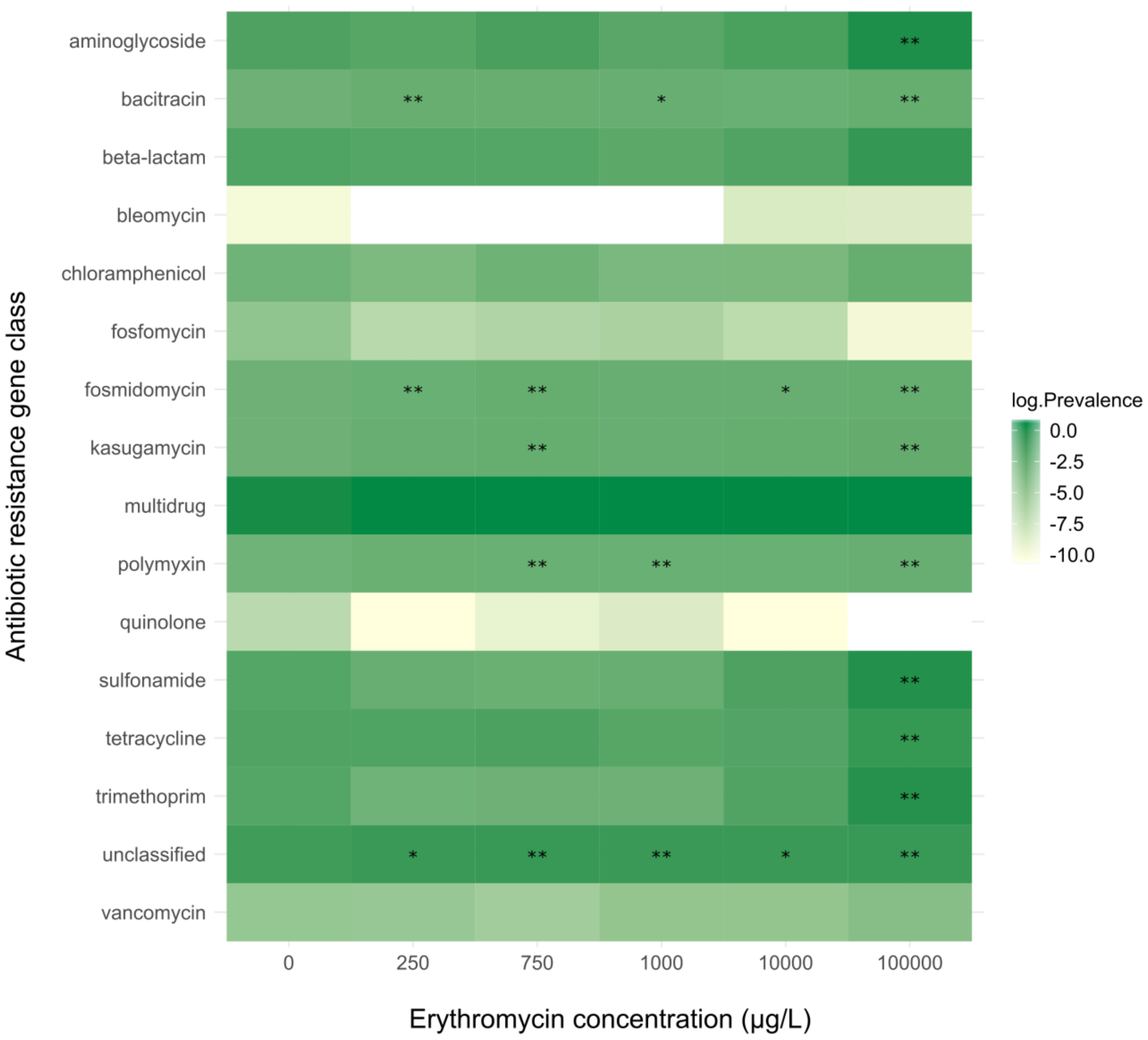

Supplementary Figure 23: Co-selection for antibiotic resistance gene classes by erythromycin. Log of the prevalence of resistance genes (normalized to 16S rRNA copy number) is represented here. * = significance to 90% confidence. ** = significance to 95% confidence.

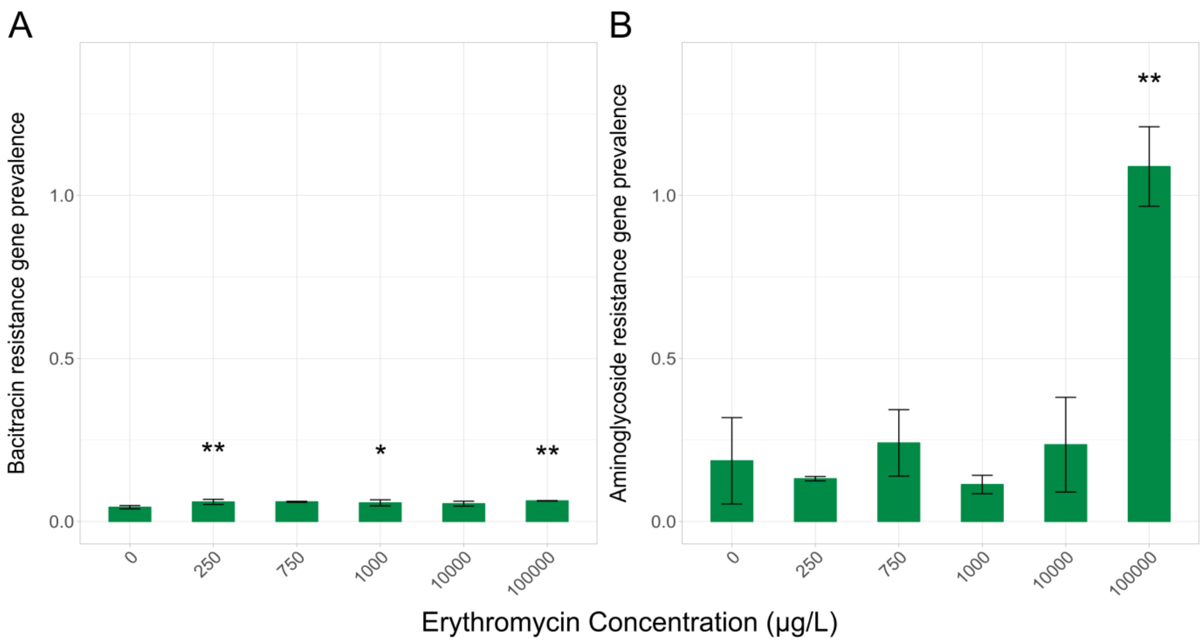

Supplementary Figure 24A: Bacitracin resistance gene prevalence as a function of erythromycin concentration. 24B: Aminoglycoside resistance gene prevalence as a function of erythromycin concentration. * = significance to 90% confidence. ** = significance to 95% confidence. Error bars represent standard error. Although the statistical analysis deems prevalence of bacitracin resistance to be significantly higher at 250 μg/L of erythromycin, in comparison to the no antibiotic control, it does not appear as if a dose dependent result is occurring (as is observed for aminoglycoside resistance). It is possible that variation in bacitracin resistance gene prevalence at the beginning of the experiment has led to a variation in the samples from the end of the experiment.

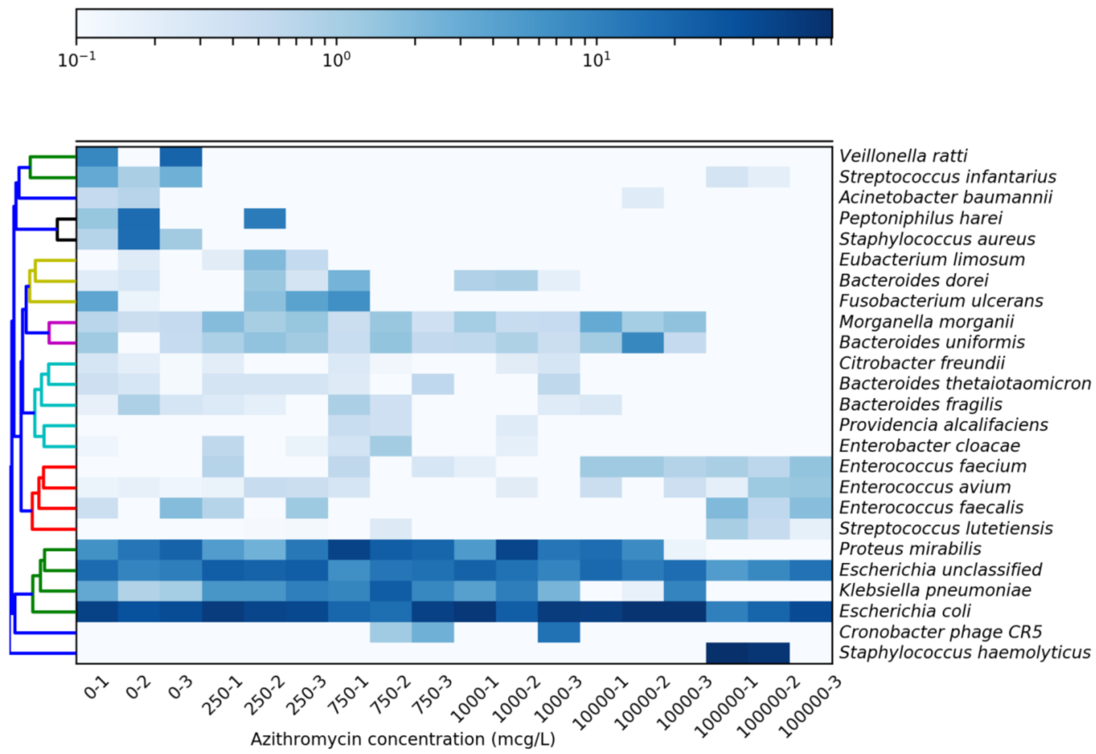

Supplementary Figure 25: Community structure as a function of azithromycin concentration. Graph displays top 25 most abundant species. Three replicates are represented for each concentration (e.g. 0 – 1 = 0 µg/L, replicate 1).

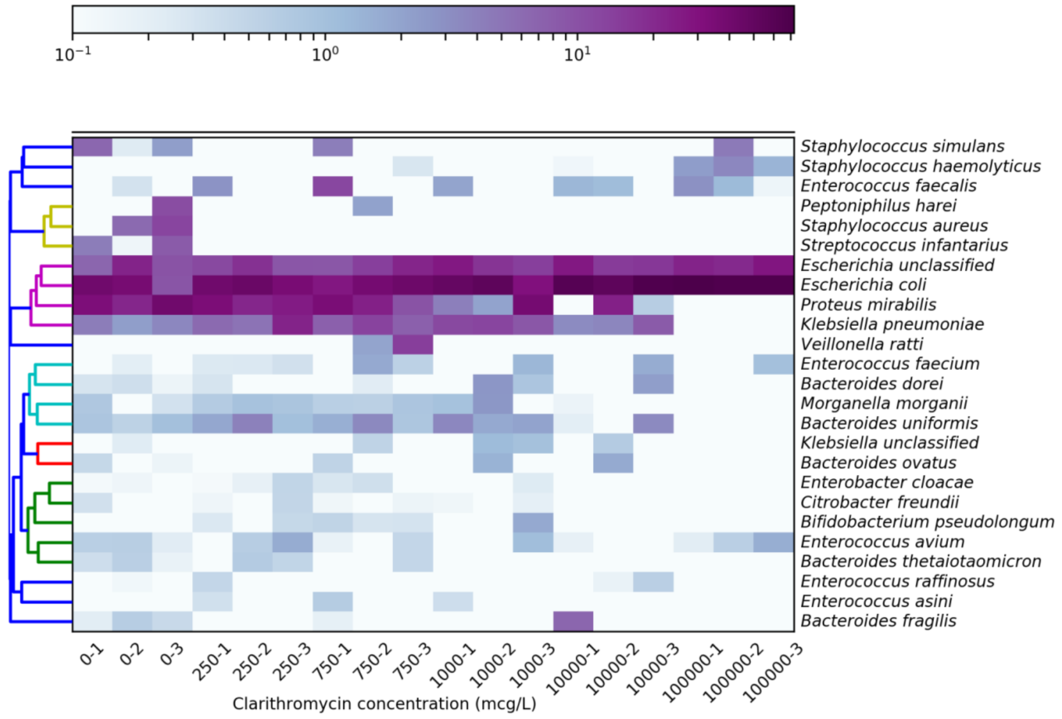

Supplementary Figure 26: Community structure as a function of clarithromycin concentration. Graph displays top 25 most abundant species. Three replicates are

represented for each concentration (e.g. 0 – 1 = 0 µg/L, replicate 1).

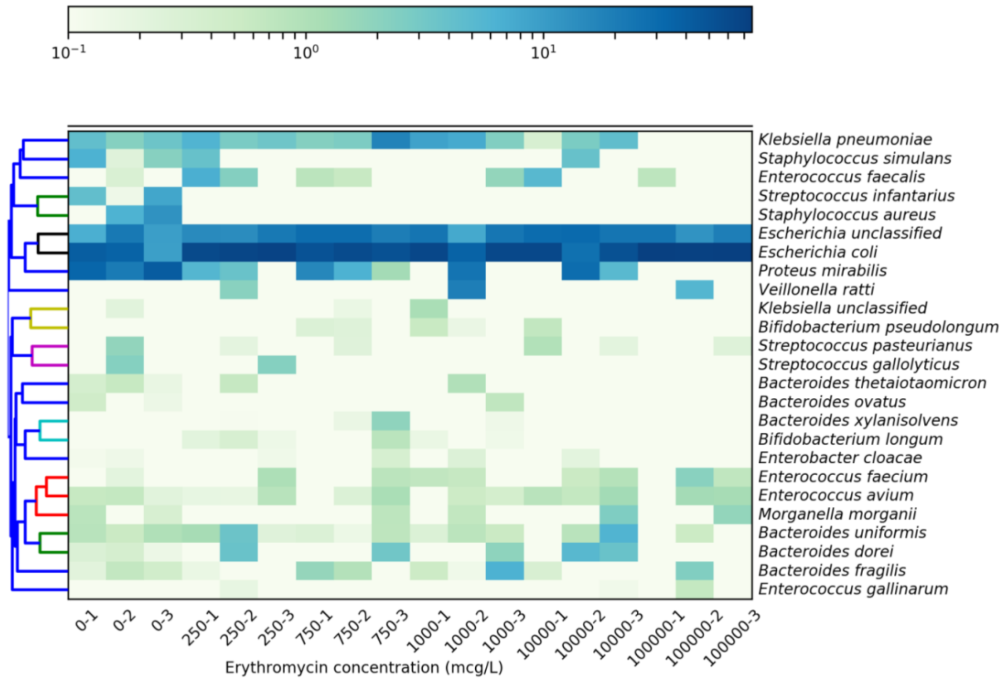

Supplementary Figure 27: Community structure as a function of erythromycin concentration. Graph displays top 25 most abundant species. Three replicates are represented for each concentration (e.g. 0 – 1 = 0 µg/L, replicate 1).

Assessing the selective potential of ciprofloxacin

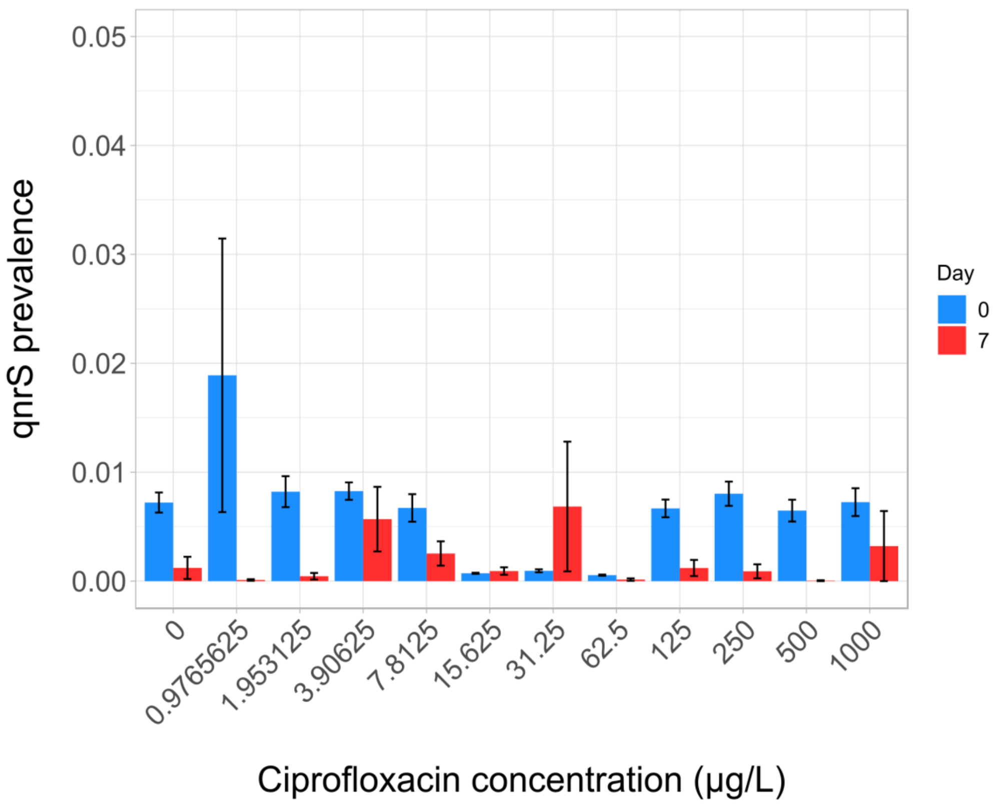

Supplementary Figure 28: *QnrS* as a function of ciprofloxacin concentration. No significant selection is seen for *qnrS* at any concentrations of ciprofloxacin. Error bars represent standard error.

A comparison of *in vitro* assays for determining MSCs

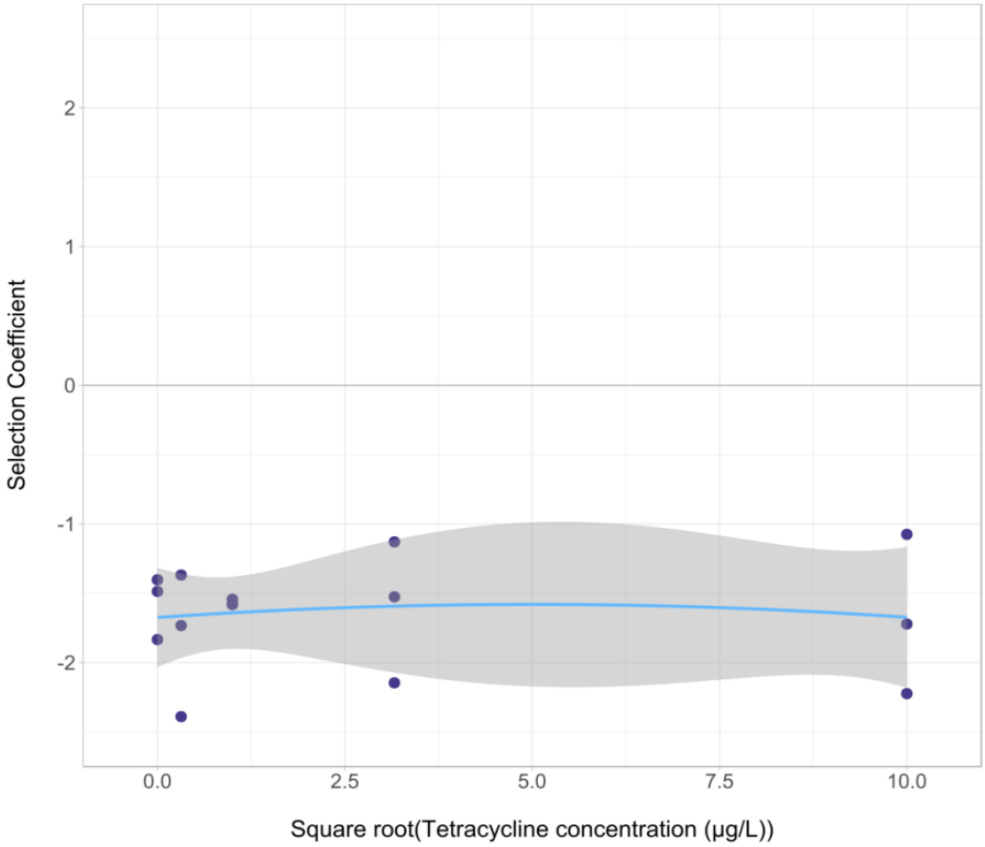

Supplementary Figure 29: Selection coefficient of *tetG* in the presence of tetracycline. An MSC cannot be determined for *tetG* as the line of best fit (polynomial order 2, $y = -1.7 + 0.038x - 0.0038x^2$, $R^2 = 0.007067$) never crosses the x axis. The square root of the tetracycline concentrations has been plotted here and represents the absolute concentration values of 0, 0.1, 1, 10 and 100 µg/L.

Supplementary Tables:

Location	AZ	CLA	ERY	ERY-H_2O	Reference
Grand Island, Nebraska	0.6695	-	-	-	Bartelt-Hunt et al. 2009
Columbus, Nebraska	0.0635	-	-	-	Bartelt-Hunt et al. 2009
Lincoln, Nebraska	1.5467	-	-	-	Bartelt-Hunt et al. 2009
Hastings, Nebraska	0.2835	-	-	-	Bartelt-Hunt et al. 2009
Omaha, Nebraska	0.6904	-	-	-	Bartelt-Hunt et al. 2009
Chivasso, N. Italy	-	0.0203	0.0159	-	Calamari et al. 2003

Mezzano, N. Italy	-	0.0012	0.0039	-	Calamari et al. 2003
Boscone, N. Italy	-	0.0016	0.0032	-	Calamari et al. 2003
Pacenza, M. Italy	-	0.0034	0.0046	-	Calamari et al. 2003
Cremona, N. Italy	-	0.0005	0.0014	-	Calamari et al. 2003
Casalmaggiore, N. Italy	-	0.0008	0.0014	-	Calamari et al. 2003
Pieve Saliceto, N. Italy	-	0.0017	0.0028	-	Calamari et al. 2003
Parco Lambro, N. Italy	-	0.0083	0.0045	-	Calamari et al. 2003
Colorado (2)	-	0.04	0.021	-	Ferrer et al. 2010
Colorado (3)	-	0.172	1.2	-	Ferrer et al. 2010
Colorado (1)	-	-	0.052	-	Ferrer et al. 2010
Colorado (2)	0.005	0.005	0.007	-	Ferrer et al. 2010
Colorado (1)	-	0.01	-	-	Ferrer et al. 2010
Ebro River Basin – Vallas	0.068	-	-	-	Gros et al. 2007
Ebro River Basin – Ebro	0.016	-	0.034	-	Gros et al. 2007
Ebro River Basin – Iregua	0.009	-	0.029	-	Gros et al. 2007
Ebro River Basin – Arga	0.022	-	0.037	-	Gros et al. 2007
Ebro River Basin – Ebro	0.023	-	0.071	-	Gros et al. 2007
Ebro River Basin – Segre	0.017	-	0.021	-	Gros et al. 2007

Ebro River Basin – Ebro	0.014	-	0.044	-	Gros et al. 2007
River Danube, Serbia	0.055	-	-	-	Grujić et al. 2009
River Tamis, Serbia	0.036	-	-	-	Grujić et al. 2009
Lake Ocaga, Serbia	0.081	-	-	-	Grujić et al. 2009
Belgrade, Serbia	0.025	-	-	-	Grujić et al. 2009
Belgrade, Serbia	0.14	-	-	-	Grujić et al. 2009
River Sava, Serbia	0.15	-	-	-	Grujić et al. 2009
Lake Wannsee, Germany	-	0.0089	-	-	Heberer et al. 2008
Germany	-	0.24	-	2.5	Hirsch et al. 1999
Germany	-	-	-	0.15	Hirsch et al. 1999
Wangyan River, China	-	-	-	0.0678	Jiang et al. 2014
River Taff, Trefforest Estate	-	-	-	0.0195	Kasprzyk-Hordern et al. 2007
River Taff, Cardiff	-	-	-	0.0075	Kasprzyk-Hordern et al. 2007
River Taff, Brecon Beacons	-	-	-	0.003	Kasprzyk-Hordern et al. 2008
River Taff, Merthyr Tydfil	-	-	-	0.001	Kasprzyk-Hordern et al. 2008
River Taff, Abercynon	-	-	-	0.007	Kasprzyk-Hordern et al. 2008
River Taff, Pontypridd	-	-	-	0.091	Kasprzyk-Hordern et al. 2008

River Taff, Tefforest Estate	-	-	-	0.061	Kasprzyk-Hordern et al. 2008
River Taff, Cardiff	-	-	-	0.08	Kasprzyk-Hordern et al. 2008
Cilfynydd, Wales	-	-	-	1.152	Kasprzyk-Hordern et al. 2009
Coslech	-	-	-	0.019	Kasprzyk-Hordern et al. 2009
River Taff, Abercynon	-	-	-	0.004	Kasprzyk-Hordern et al. 2009
River Taff, Pontypridd	-	-	-	0.052	Kasprzyk-Hordern et al. 2009
River Ely, Peterson-super	-	-	-	0.005	Kasprzyk-Hordern et al. 2009
Iowa (1)	-	-	-	0.22	Kolpin et al. 2004
Iowa (2)	-	-	-	0.02	Kolpin et al. 2004
Southern Ontario	-	-	0.0056	-	Lissemore et al. 2006
Ebro, Spain	0.0046	0.0121	-	-	López-Serna et al. 2012
Ebro tributaries, Spain	0.0044	0.022	-	0.0007	López-Serna et al. 2012
Umgeni River, S Africa (North outlet)	-	-	0.24	-	Matongo et al. 2015
Lausanne, Switzerland	0.96	1	-	-	Morasch et al. 2010
Llobregat River Basin	-	-	0.03	-	Muñoz et al. 2009
Tone River, Japan – mainstream	0.008	0.012	-	-	Nakada et al. 2007

Tone River, Japan – tributary	0.0065	0.013	-	-	Nakada et al. 2007
Tone River, Japan	0.165	0.568	-	-	Nakada et al. 2007
River Lein, Germany	-	0.077	0.022	-	Nödler et al. 2010
Baltic Sea, Usedom	-	0.014	-	-	Nödler et al. 2010
Not stated	-	0.52	0.173	-	Nödler et al. 2010
Llobregat, Spain	-	-	0.3625	-	Osorio et al. 2012
Llobregat, Spain			0.107		Osorio et al. 2012
Ebro, Spain	-	-	0.071	-	Osorio et al. 2012
Yangtze Estuary, China	-	-	-	0.0896	Shi et al. 2014
Thames, Oxford	0.03	0.092	0.236	-	Singer et al. 2014
Thames, Benson	0.034	0.05	0.244	-	Singer et al. 2014
Northwest Ohio (1)	-	0.702	-	-	Spongberg & Witter 2008
Northwest Ohio (2)	-	0.6106	-	-	Spongberg & Witter 2008
Jianhan Plain, China	-	-	-	4	Tong et al. 2014
Jianghan Plain, China	-	-	-	2.3	Tong et al. 2014
Pearl River Delta, S China (1)	-	-	0.43	-	Xu et al. 2007a
Pearl River Delta, S China (2)	-	-	2.054	-	Xu et al. 2007a
Pearl River Delta, S China (3)	-	-	0.216	-	Xu et al. 2007a

Pearl River Delta, S China (4)	-	-	0.259	-	Xu et al. 2007a
Victoria Harbour, Hong Kong	-	-	-	0.00335	Xu et al. 2007b
Pearl River (high water season)	-	-	-	0.03	Xu et al. 2007b
Pearl River (low water season)	-	-	-	0.46	Xu et al. 2007b
Jianghan Plain, China (winter)	-	-	0.51	-	Yao et al. 2015
Jianghan Plain, China (spring)	-	-	2.42	-	Yao et al. 2015
Yangtze Estuary, China	-	-	0.0076	-	Zhao et al. 2015
River Po, Italy	-	0.0016	0.0032	-	Zuccato et al. 2010
River Lambro, Italy	-	0.0083	0.0045	-	Zuccato et al. 2010
River Po, Mezzana Corti, Italy	-	0.0018	0.0008	0.00166	Zuccato et al. 2010
River Po, Monticelli Pv, Italy	-	0.0009	0.0035	0.00427	Zuccato et al. 2010
River Po, Piacenza, Italy	-	0.0022	0.0046	0.00531	Zuccato et al. 2010
River Po, Cremona, Italy	-	0.0019	0.0028	0.00363	Zuccato et al. 2010
River Arno, Rignano sull’Arno, Italy		0.0067	0.0039	0.01396	Zuccato et al. 2010
River Arno, Limite sull’Arno, Italy		0.0166	0.0029	0.00968	Zuccato et al. 2010

River Arno, Castelfranco, Italy		0.0336	0.0068	0.01729	Zuccato et al. 2010
River Arno, Pisa, Italy		0.0448	0.0081	0.03052	Zuccato et al. 2010
Milan, Italy	-	0.104	0.034	-	Zuccato et al. 2010
Varese, Italy	-	0.052	0.027	-	Zuccato et al. 2010
Lugano, Italy	-	0.437	0.059	-	Zuccato et al. 2010
Como, Italy	-	0.5	0.0065	0.017	Zuccato et al. 2010
Italy (urban wastewater)	-	0.0181	0.0474	-	Zuccato et al. 2010

Supplementary Table 1: Measured environmental concentrations of macrolide antibiotics. All concentrations are in µg/L. AZ = azithromycin, CLA = clarithromycin, ERY = erythromycin and ERY-H2O = erythromycin-H2O. A list of full references can be found below in the "Supplementary References" section.

Supplementary References:

Bartelt-Hunt, S.L. et al., 2009. The occurrence of illicit and therapeutic pharmaceuticals in wastewater effluent and surface waters in Nebraska. Environmental Pollution, 157(3), pp.786–791.

Calamari, D. et al., 2003. Strategic survey of therapeutic drugs in the rivers Po and lambro in Northern Italy. Environmental Science and Technology, 37(7), pp.1241–1248.

Ferrer, I., Zweigenbaum, J.A. & Thurman, E.M., 2010. Analysis of 70 Environmental Protection Agency priority pharmaceuticals in water by EPA Method 1694. Journal of Chromatography A, 1217(36), pp.5674–5686.

Gros, M., Petrovic, M. & Barceló, D., 2007. Wastewater treatment plants as a pathway for aquatic contamination by pharmaceuticals in the Ebro river basin (northeast Spain). Environmental Toxicology and Chemistry, 26(8), pp.1553–1562.

Grujić, S., Vasiljević, T. & Laušević, M., 2009. Determination of multiple pharmaceutical classes in surface and ground waters by liquid chromatography-ion trap-tandem mass spectrometry. Journal of Chromatography A, 1216(25), pp.4989–5000.

Heberer, T. et al., 2008. Behaviour and redox sensitivity of antimicrobial residues during bank filtration. Chemosphere, 73(4), pp.451–460.

Hirsch, R. et al., 1999. Occurrence of antibiotics in the aquatic environment. Science of the Total Environment, 225(1–2), pp.109–118.

Jiang, Y. et al., 2014. Distribution and ecological risk of antibiotics in a typical effluent-receiving river (Wangyang River) in north China. Chemosphere, 112, pp.267–274.

Kasprzyk-Hordern, B., Dinsdale, R.M. & Guwy, A.J., 2007. Multi-residue method for the determination of basic/neutral pharmaceuticals and illicit drugs in surface water by solid-phase extraction and ultra performance liquid chromatography-positive electrospray ionisation tandem mass spectrometry. Journal of Chromatography A, 1161(1–2), pp.132–145.

Kasprzyk-Hordern, B., Dinsdale, R.M. & Guwy, A.J., 2008. The occurrence of pharmaceuticals, personal care products, endocrine disruptors and illicit drugs in surface water in South Wales, UK. Water Research, 42(13), pp.3498–3518.

Kasprzyk-Hordern, B., Dinsdale, R.M. & Guwy, A.J., 2009. The removal of pharmaceuticals, personal care products, endocrine disruptors and illicit drugs during wastewater treatment and its impact on the quality of receiving waters. Water Research, 43(2), pp.363–380.

Kolpin, D.W. et al., 2004. Urban contribution of pharmaceuticals and other organic wastewater contaminants to streams during differing flow conditions. Science of the Total Environment, 328(1–3), pp.119–130.

Lissemore, L. et al., 2006. An exposure assessment for selected pharmaceuticals within a watershed in Southern Ontario. Chemosphere, 64(5), pp.717–729.

López-Serna, R., Petrović, M. & Barceló, D., 2012. Occurrence and distribution of multi-class pharmaceuticals and their active metabolites and transformation products in the Ebro River basin (NE Spain). Science of the Total Environment, 440, pp.280–289.

Matongo, S. et al., 2015. Occurrence of selected pharmaceuticals in water and sediment of Umgeni River, KwaZulu-Natal, South Africa. Environmental Science and Pollution Research, 22(13), pp.10298–10308.

Morasch, B. et al., 2010. Occurrence and fate of micropollutants in the Vidy Bay of Lake Geneva, Switzerland. Part II: Micropollutant removal between wastewater and raw drinking water. Environmental Toxicology and Chemistry, 29(8), pp.1658–1668.

Muñoz, I. et al., 2009. Bridging levels of pharmaceuticals in river water with biological community structure in the Llobregat River basin (northeast Spain). Environmental Toxicology and Chemistry / SETAC, 28(12), pp.2706–14.

Nakada, N. et al., 2007. Occurrence of 70 pharmaceutical and personal care products in Tone River basin in Japan. Water Science and Technology, 56(12), pp.133–140.

Nödler, K. et al., 2010. Development of a multi-residue analytical method, based on liquid chromatography-tandem mass spectrometry, for the simultaneous determination of 46 micro-contaminants in aqueous samples. Journal of Chromatography A, 1217(42), pp.6511–6521.

Osorio, V. et al., 2012. Occurrence and modeling of pharmaceuticals on a sewage-impacted Mediterranean river and their dynamics under different hydrological conditions. Science of the Total Environment, 440, pp.3–13.

Shi, X. et al., 2014. Application of passive sampling in assessing the occurrence and risk of antibiotics and endocrine disrupting chemicals in the Yangtze Estuary, China. Chemosphere, 111, pp.344–351.

Singer, A.C. et al., 2014. Intra- and inter-pandemic variations of antiviral, antibiotics and decongestants in wastewater treatment plants and receiving rivers. PLoS ONE, 9(9).

Spongberg, A.L. & Witter, J.D., 2008. Pharmaceutical compounds in the wastewater process stream in Northwest Ohio. Science of the Total Environment, 397(1–3), pp.148–157.

Tong, L. et al., 2014. Occurrence of antibiotics in the aquatic environment of Jianghan Plain, central China. Science of the Total Environment, 497–498, pp.180–187.

Xu, W. et al., 2007a. Occurrence and elimination of antibiotics at four sewage treatment plants in the Pearl River Delta (PRD), South China. Water Research, 41(19), pp.4526–4534.

Xu, W. et al., 2007b. Determination of selected antibiotics in the Victoria Harbour and the Pearl River, South China using high-performance liquid chromatography-electrospray ionization tandem mass spectrometry. Environmental Pollution, 145(3), pp.672–679.

Yao, L. et al., 2015. Seasonal variation of antibiotics concentration in the aquatic environment: A case study at Jianghan Plain, central China. Science of the Total Environment, 527–528, pp.56–64.

Zhao, H., Zhou, J.L. & Zhang, J., 2015. Tidal impact on the dynamic behavior of dissolved pharmaceuticals in the Yangtze Estuary, China. Science of the Total Environment, 536, pp.946–954.

Zuccato, E. et al., 2010. Source, occurrence and fate of antibiotics in the Italian aquatic environment. Journal of Hazardous Materials, 179(1–3), pp.1042–1048.
