# Supplementary material for: Evolution of antibiotic resistance at low antibiotic concentrations including selection below the minimal selective concentration
Source: Commun Biol. 2020 Sep 3;3:467. doi: 10.1038/s42003-020-01176-w (PMC7471295; doi:10.1038/s42003-020-01176-w)
Supplement: Supplementary file 3 — Reporting Summary [file 42003_2020_1176_MOESM3_ESM.pdf]

nature research

Corresponding author(s): William Gaze

Last updated by author(s): Jun 25, 2020

Reporting Summary

Nature Research wishes to improve the reproducibility of the work that we publish. This form provides structure for consistency and transparency in reporting. For further information on Nature Research policies, see our Editorial Policies and the Editorial Policy Checklist.

Statistics

For all statistical analyses, confirm that the following items are present in the figure legend, table legend, main text, or Methods section.

n/a	Confirmed	
☐	☒	The exact sample size (n) for each experimental group/condition, given as a discrete number and unit of measurement
☐	☒	A statement on whether measurements were taken from distinct samples or whether the same sample was measured repeatedly
☐	☒	The statistical test(s) used AND whether they are one- or two-sided<br>*Only common tests should be described solely by name; describe more complex techniques in the Methods section.*
☐	☒	A description of all covariates tested
☐	☒	A description of any assumptions or corrections, such as tests of normality and adjustment for multiple comparisons
☐	☒	A full description of the statistical parameters including central tendency (e.g. means) or other basic estimates (e.g. regression coefficient) AND variation (e.g. standard deviation) or associated estimates of uncertainty (e.g. confidence intervals)
☐	☒	For null hypothesis testing, the test statistic (e.g. F, t, r) with confidence intervals, effect sizes, degrees of freedom and P value noted<br>*Give P values as exact values whenever suitable.*
☒	☐	For Bayesian analysis, information on the choice of priors and Markov chain Monte Carlo settings
☒	☐	For hierarchical and complex designs, identification of the appropriate level for tests and full reporting of outcomes
☐	☒	Estimates of effect sizes (e.g. Cohen's d, Pearson's r), indicating how they were calculated

Our web collection on statistics for biologists contains articles on many of the points above.

Software and code

Policy information about availability of computer code

Data collection	An Applied Biosystems StepOne machine was used to collect the qPCR data.<br>A HiSeq 2500 was used for sequence samples.
Data analysis	R studio was used to undertake the statistical analysis. Packages installed were dunn.test and multcomp.<br>To check quality of the metagenome sequence files: FastQC (https://github.com/s-andrews/FastQC) and MultiQC (https://github.com/ewels/MultiQC) were used.<br>For 16S analysis of the metagenome sequence data: FLASH2 was used to pair the sequence reads (https://github.com/dstreett/FLASH2), 16S identity was asigned using metaphlan2 (https://github.com/biobakery/biobakery/wiki/metaphlan2#create-taxonomic-profiles) and diversity plots were created using hclust2 (https://github.com/SegataLab/hclust2).<br>ARGs-OAP v2 was used to undertake the metagenome analysis for the resistance genes (https://github.com/xiaole99/ARGs-OAP-v2.0).

For manuscripts utilizing custom algorithms or software that are central to the research but not yet described in published literature, software must be made available to editors and reviewers. We strongly encourage code deposition in a community repository (e.g. GitHub). See the Nature Research guidelines for submitting code & software for further information.

Data

Policy information about availability of data

All manuscripts must include a data availability statement. This statement should provide the following information, where applicable:

- Accession codes, unique identifiers, or web links for publicly available datasets
- A list of figures that have associated raw data
- A description of any restrictions on data availability

The datasets associated with Figures 1 – 6 are included in this published article as a Supplementary Data file. Metagenome sequence files have been deposited in the European Nucleotide Archive. Accession number: PRJEB38942.

Field-specific reporting

Please select the one below that is the best fit for your research. If you are not sure, read the appropriate sections before making your selection.

☐ Life sciences ☐ Behavioural & social sciences ☒ Ecological, evolutionary & environmental sciences

For a reference copy of the document with all sections, see nature.com/documents/nr-reporting-summary-flat.pdf

Ecological, evolutionary & environmental sciences study design

All studies must disclose on these points even when the disclosure is negative.

Study description	The selective potential of 5 antibiotic compounds, from 3 classes were investigated.<br>Raw wastewater inoculum was grown in isosensitest broth at 37oC at multiple concentrations of each antibiotic. For the main macrolide datasets and the ciprofloxacin dataset, 5 replicates per concentration were used. This number of replication was chosen based on previous work by Murray et al. 2018. For the macrolide range finding experiments and tetracycline datasets, 3 biological replicates were used. DNA was extracted from samples taken from evolution experiments on day 0 and day 7. qPCR was undertaken on a range of resistance gene targets, the intI1 gene and the 16S rRNA gene using 2 technical replicates. Metagenome and plating analysis was also undertaken on the macrolide samples.
Research sample	The research sample inoculum was raw wastewater collected from a wastewater treatment plant serving Falmouth and Penryn, UK. Day 0 and Day 7 samples taken were taken pre (0) and post (7) experimental evolution.
Sampling strategy	One grab sample was taken of the raw wastewater inoculum to ensure consistency between experiments.
Data collection	qPCR was used to collect abundance of resistance gene, intI1 gene or 16s rRNA gene. IS exported the data once ensuring it met the quality control checks (efficiency, R0, melt curves). This data was then stored in excel and analysed using R.
Timing and spatial scale	Only one grab sample was taken for the wastewater inoculum and was frozen in aliquots at -80oC until required.<br>Sampling during the evolution experiments occurred at Day 0 (before overnight culturing) and Day 7 (after 7 serial overnight cultures).
Data exclusions	One replicate has been removed from the Figure 1B as it is a high outlier (day 7, 250 ug/L).<br><br>In the Supplementary information a small number of outliers are removed from various experiments. These are indicated in the figure legends.
Reproducibility	For the macrolide antibiotics, consistency between results was observed between range finding experiments (Supplementary information) and full experiments (Main text).<br>The data taken for tetracycline and ciprofloxacin did not undergo multiple experiments but produced similar results to previously published data (Lundstrom et al. 2016 and Kraupner et al. 2018).
Randomization	Randomization was not relevant to this study. The same wastewater inoculum was subjected to multiple concentrations of various antibiotics.
Blinding	Blinding was not relevant to this kind of study design.

Did the study involve field work? ☐ Yes ☒ No

Reporting for specific materials, systems and methods

We require information from authors about some types of materials, experimental systems and methods used in many studies. Here, indicate whether each material, system or method listed is relevant to your study. If you are not sure if a list item applies to your research, read the appropriate section before selecting a response.

Materials & experimental systems

n/a	Involved in the study
☒	☐ Antibodies
☒	☐ Eukaryotic cell lines
☒	☐ Palaeontology and archaeology
☒	☐ Animals and other organisms
☒	☐ Human research participants
☒	☐ Clinical data
☒	☐ Dual use research of concern

Methods

n/a	Involved in the study
☒	☐ ChIP-seq
☒	☐ Flow cytometry
☒	☐ MRI-based neuroimaging
