# Supplementary material for: Evolution of antibiotic resistance at low antibiotic concentrations including selection below the minimal selective concentration
Source: Commun Biol. 2020 Sep 3;3:467. doi: 10.1038/s42003-020-01176-w (PMC7471295; doi:10.1038/s42003-020-01176-w)
Supplement: Supplementary file 4 — Description of Additional Supplementary Files [file 42003_2020_1176_MOESM4_ESM.pdf]

Description of Additional Supplementary Files

File Name: **Supplementary Data 1**
Description: the source data for figures
